# Linking gene dynamics to vascular hyperplasia – Toward a predictive model of vein graft adaptation

**DOI:** 10.1371/journal.pone.0187606

**Published:** 2017-11-30

**Authors:** Stefano Casarin, Scott A. Berceli, Marc Garbey

**Affiliations:** 1 LASIE UMR 7356 CNRS, University of La Rochelle, La Rochelle, France; 2 Houston Methodist Hospital Research Institute, Houston, Texas, United States of America; 3 Department of Surgery, Houston Methodist Hospital, Houston, Texas, United States of America; 4 Malcom Randall VAMC, Gainesville, Florida, United States of America; 5 Department of Surgery, University of Florida, Gainesville, Florida, United States of America; Politecnico di Milano, ITALY

## Abstract

Reductionist approaches, where individual pieces of a process are examined in isolation, have been the mainstay of biomedical research. While these methods are effective in highly compartmentalized systems, they fail to account for the inherent plasticity and non-linearity within the signaling structure. In the current manuscript, we present the computational architecture for tracking an acute perturbation in a biologic system through a multiscale model that links gene dynamics to cell kinetics, with the overall goal of predicting tissue adaptation. Given the complexity of the genome, the problem is made tractable by clustering temporal changes in gene expression into unique patterns. These cluster elements form the core of an integrated network that serves as the driving force for the response of the biologic system. This modeling approach is illustrated using the clinical scenario of vein bypass graft adaptation. Vein segments placed in the arterial circulation for treatment of advanced occlusive disease can develop an aggressive hyperplastic response that narrows the lumen, reduces blood flow, and induces *in situ* thrombosis. Reducing this hyperplastic response has been a long-standing but unrealized goal of biologic researchers in the field. With repeated failures of single target therapies, the redundant response pathways are thought to be a fundamental issue preventing progress towards a solution. Using the current framework, we demonstrate how theoretical genomic manipulations can be introduced into the system to shift the adaptation to a more beneficial phenotype, where the hyperplastic response is mitigated and the risk of thrombosis reduced. Utilizing our previously published rabbit vein graft genomic data, where grafts were harvested at time points ranging from 2 hours to 28 days and under differential flow conditions, and a customized clustering algorithm, five gene clusters that differentiated the low flow (i.e., pro-hyperplastic) from high flow (i.e., anti-hyperplastic) response were identified. The current analysis advances these general associations to create a model that identifies those genes sets most likely to be of therapeutic benefit. Using this approach, we examine the range of potential opportunities for intervention via gene cluster over-expression or inhibition, delivered in isolation or combination, at the time of vein graft implantation.

## Introduction

While endovascular interventions via angioplasty and/or stent placement have defined roles in the treatment of arterial occlusive pathologies, bypass grafting remains the most effective therapy to re-establish flow in the setting of advanced coronary and peripheral lesions [1–4].

Providing a pathway to shunt blood around these segmental regions of high-grade stenosis or occlusion, the long-term success of these interventions is implicitly linked to the durability of these conduits to provide an unobstructed pathway for flow. While a variety of biomaterials has been developed for this purpose, autologous vein remains the conduit of choice for these procedures. Although being conceptually the ideal conduit, failure rates remains unacceptably high, approaching 40% within one year following implantation [5,6].

Implicit in the creation of a vein graft is the transposition of this conduit from a low pressure/continuous flow regime to a high pressure/pulsatile flow environment. This initiates a series of adaptation and repair mechanisms that are critical in maintaining structural stability in the face of these more extreme hemodynamics [4,7,8]. While this arterialization process, characterized by thickening of the wall and expansion of the lumen, provides a normalization of the biomechanical forces to a more physiologic level, the biologic processes that regulate this adaptation can overcompensate, leading to an aggressive hyperplastic response and narrowing of the lumen. This maladaptive phenotype, and the resulting stenotic lesion, results in a significant reduction in blood flow through the graft and failure secondary to in situ thrombosis [9–13].

Attempts to develop targeted pharmacologic therapies to mitigate this aggressive hyperplastic response and improve vein graft outcomes have been unsuccessful [5,6]. Our group and others [14–16] have postulated that the redundancy among the pathways that regulate this maladaptive response undermines the success of a “single-bulleted” approach, and multiple targeted therapies at critical stages in the disease process are required for a successful outcome. The challenge remains to identify those cornerstone elements that can be manipulated to alter the trajectory of this response.

Investigations has previously shown that changes in the hemodynamics environment within blood vessel are perceived at the genomic level [17,18]. Levering this concept, the current manuscript details the methodology to utilize high-throughput genomic data to create a multiscale model of vein graft adaptation. The rationale is to offer a general mathematical construct that can simulate the outcome of targeted gene therapies and reduce the complexity from millions of possible combinations to few hundred of potential ones. Within the proposed framework, unique temporal patterns of gene expression are quantitatively linked to their effect on cell and matrix kinetics, and ultimately their impact on graft architecture. The resulting predictive model provides a tool for the *in silico* exploration of the connection between gene regulatory networks and the adaptive response of grafts, identifying key gene sets that can be manipulated to mitigate the maladaptive remodeling response following vein graft implantation. The current version of the model wants to serve as a modular framework. Our plan is to keep improving the model and to further narrow the gene therapy target with *in vivo* validation.

## Materials and methods

### Multiscale model

**Fig 1:**
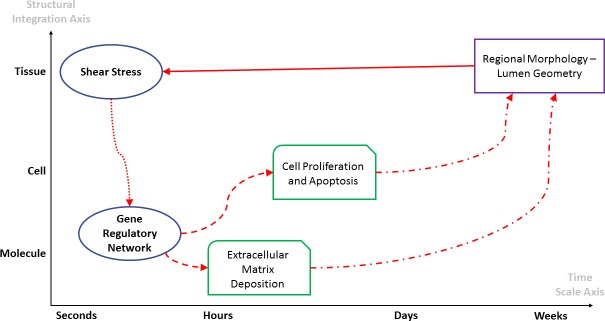
System biology approach. The vein graft arterialization process is described by a loop of interdependent events, where the dynamic interplay between physical forces and gene network regulates the early graft remodeling.

In order to understand the complex interplay of all the elements influencing the vascularization of the graft, we chose a systems biology approach that puts an emphasis on understanding the intervening components and on providing predictive models to anticipate the final outcome [19,20]. Fundamental to the systems biology approach is the understanding of the existence of a critical link between the system, in our case the vein graft, and the environment. Perturbations of the environment influence the structure and the function of the system, which impacts the environment itself creating a feedback loop between system and environment. This interaction may lead to a relative homeostasis, where variations in the environment and in the system converge to a stable phenotype, but also it may result in a dynamic instability if one side of the loop is not properly balanced by the other. Early vein graft remodeling is the perfect example of how the balance between system and environment may drive the surgical outcome toward a stable phenotype, or toward the failure of the procedure. The current model is based on the concept of a direct link between hemodynamics and transcriptional regulation as illustrated in Fig 1. The result is a highly interdependent system where local perturbations provide feedback to other elements leading the system either to a new set point, which represents the arterialized vein, or to instability, which represents the restenosis phenomenon that leads to the failure of the surgical procedure.

The environmental condition (shear stress) directs the initial working point of the gene network, which dictates the cellular and the matrix-based remodeling response of the vein. Changes at the cellular level define the local graft architecture, which directly impacts the shear stress that determines a new set point for the gene network, and consequently a new biological response of the graft.

Our multiscale model is made up of 2 distinct parts: i) a subset of a Dynamical System (DS) already developed in our previous work [21], and ii) a gene Cluster Network (CN). The first is a heuristic model derived from a conceptual diagram based on experimental observations, with the feature to be able to predict the final outcome of the vein graft arterialization, while the second is implemented as a system of Ordinary Differential Equations (ODE) that replicates both the expression and the level of mutual interconnectedness of targeted cluster of genes. The two parts are combined to form a hybrid model able to cover both the macro and the micro scale aspect of hyperplasia.

**Fig 2:**
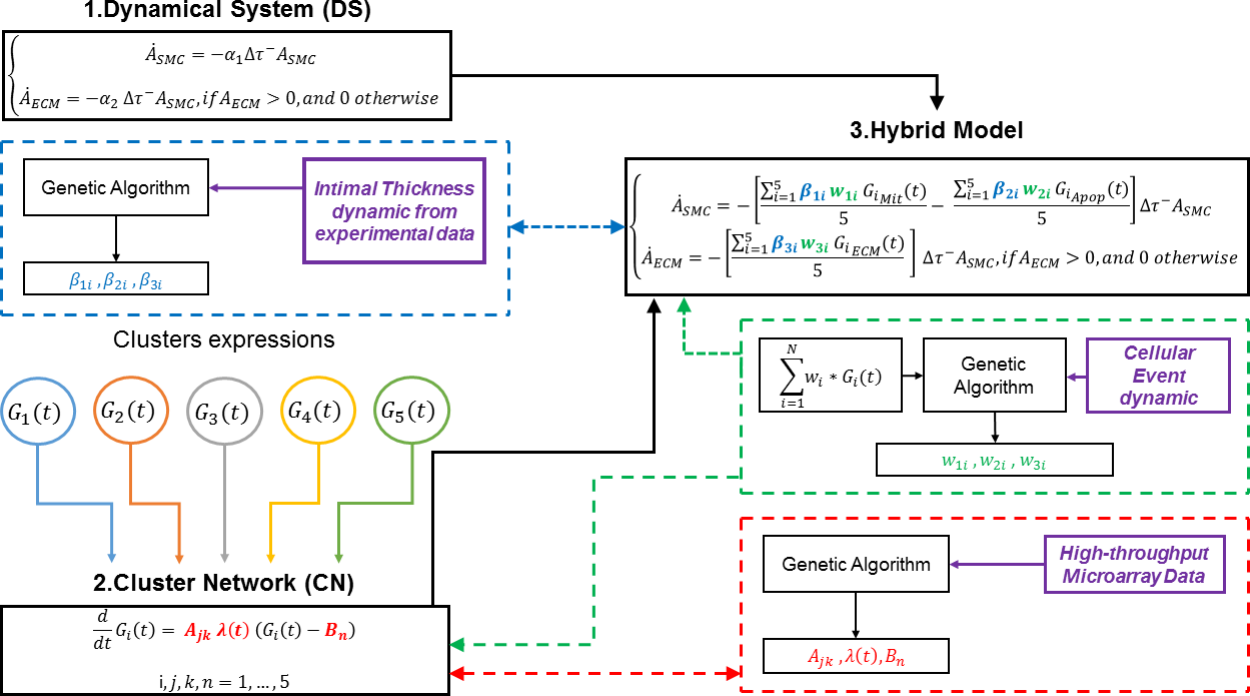
Hybrid model development step-by-step diagram. Significant clusters of expression (G_i_(t)) are organized in Cluster Network (CN) through an Ordinary Differential Equations (ODE) system, which unknowns (highlighted in bold red) are retrieved fitting the ODE system on experimental data from gene microprobes (dashed red line box). The CN is plugged into a Dynamical System (DS) that simulate the long-term vein graft healing, to create a hybrid model, characterized by 2 kinds of unknowns: in bold green the weights of each cluster on a specific cellular event, that are retrieved on the base of experimental data (dashed green line box); in bold blue the scaling factors that adjust the unite of measurement of each cluster expression into the hybrid model, also retrieved on the base of experimental data (dashed blue line box). In general, a large use of heterogenic experimental data (highlighted in bold purple) has been made at various levels through the development of the model for validation and calibration purposes.

Fig 2 details the general construct of the hybrid model, which can be summarized in five fundamental steps:

1. Construction of the Dynamical System (DS) that replicates hyperplasia (first sub-model);
2. Construction and calibration of the Cluster Network (CN), that details the expression of each single cluster of genes and the level of mutual interconnectedness among them (second sub-model);
3. Coupling of CN and DS to form the hybrid model;
4. Calibration of the different level of impact that each component of each network has on the specific biologic event (relative weights of the clusters);
5. Calibration of the hybrid model on experimental data.

The skeleton of the multiscale model is implemented as a Matlab^®^ code and also all the couplings and the validations introduced have been performed by using Matlab^®^ functions from the Optimization Toolbox. The code implemented has not been provided within the current publication, however it will be provided upon request to foster and encourage future collaborations.

#### Experimental setup

Our multiscale model was calibrated at various levels through non-linear fitting on experimental data, which were retrieved from a rabbit vein graft model, which included shear-modulation in order to examine the influence of hemodynamics on graft remodeling [22–25]. Specifically the experimental data are retrieved from our previously published work [26], where jugular veins were inserted into both the left and right common carotid arteries of the rabbit and coupled with unilateral ligation of the internal carotid. This causes a 90% reduction in flow on the ligated vein graft side, which enhances the hyperplasic response and narrows the lumen [27–31]. Grafts were harvested at multiple time points, ranging from 2 hours to 28 days, to facilitate microarray, cell and matrix kinetic, and graft morphology measurements that are needed to calibrate the model.

All data needed for the calibration and the validation of the multiscale model were retrieved from the rabbit model described [26] and they will be presented in detail at the beginning of each corresponding section.

#### 1. Dynamical System (DS)

Hyperplasia is the dominant event in the first month of graft’s adaptation [4,7] and it is primarily driven by alterations in shear stress [8,27,28,31,32]. The atherosclerotic disease progression in the venous system is indeed analogous to the one recorded for arterial system, in which local wall shearing forces have been postulated as a major regulator of vein graft adaptation [33]. Suggested by an array of animal experiments from both our group [34] and others [35] and also more recently confirmed by observations in humans [4], reductions in local wall shear have been demonstrated to be critical components leading to accelerated intimal hyperplasia development. In addition, as studied by Langille BL et al. [36] and Kohler TR et al. [37], morphological changes related to graft adaptation in the first weeks of follow-up are strongly endothelial dependent and the endothelium is strongly sensitive to shear stress [37]. Accordingly, in order to study the temporal dynamic of the hyperplastic response, we used an adapted subset of a previously developed DS [21]. The latter is a system of Ordinary Differential Equations (ODEs) designed to model and replicate the interconnectedness between shear/tensile forces, biologic processes, and morphology changes within the vein graft following the implantation. The model approximates the geometry of the graft as a straight, thick, and circumferential symmetric cylinder with internal radius R_1_ and external radius R_2_, and internal pressure P_1_ and external pressure P_2_. Accordingly, assuming a Poiseuille flow across the cylinder, the dynamic of the intima is solely led by shear stress, given by the formula:

$$\tau =\mu \frac{2U}{R_{1}},$$ (1)

where U is the maximum velocity of the blood at the centerline, and *μ* is the dynamic viscosity of the blood.

**Fig 3:**
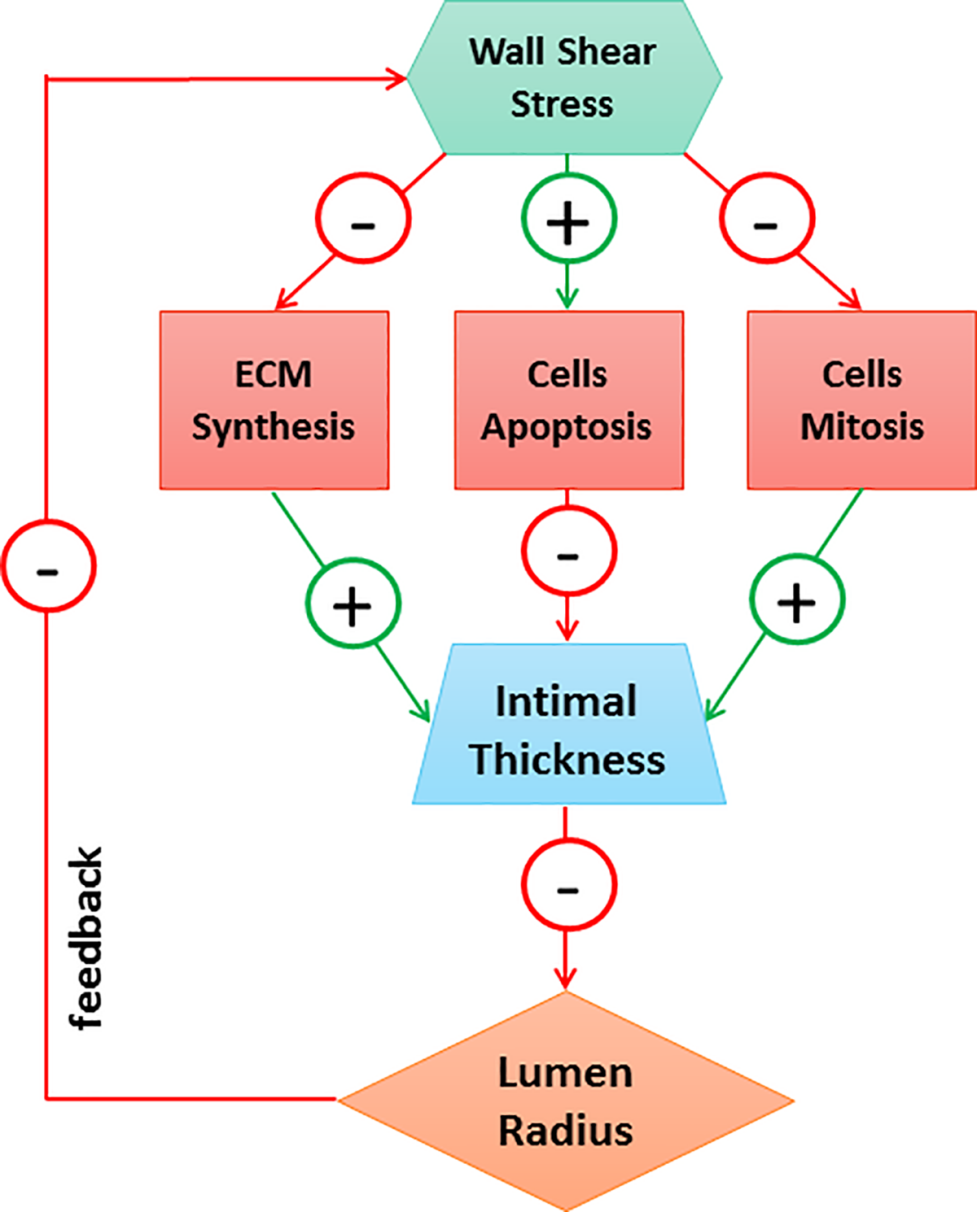
Dynamical System (DS). Conceptual scheme of the subset of DS that replicates hyperplasia during the first month of post-surgical follow-up [21].

For the purpose of this work, we extracted a new conceptual scheme, reported in Fig 3, and we based our version of the DS on it. Our sub-model of graft adaptation is fully described by the following system of ODEs:

$$\left\{ \begin{array}{c} {\dot{A}}_{SMC}=-\alpha_{1}\Delta \tau^{-}A_{SMC}\\ {\dot{A}}_{ECM}=-\alpha_{2}\;\Delta \tau^{-}A_{SMC} \end{array}.\right.$$ (2)

In (2), A_SMC_ is the cross-sectional area occupied by cellular density and A_ECM_ the area occupied by the extracellular matrix (ECM) density. Δ*τ*^−^ = min(Δ*τ*,0), is the deviation of the shear stress from its baseline, which is imposed to be negative in order to enhance cellular mitosis.

The model is indeed formulated such that a reduction in shear stress is the driving force for augmented hyperplasia. Letting *τ*_0_ be the shear stress at time t = 0 (assumed to be the time of implantation of the graft), the intimal growth rate can be expressed as a function of the difference between the mechanical condition at time t and the baseline setting (recorded at t = 0):

$$\Delta \tau =\tau (t)-\tau_{0}.$$ (3)

Assuming unitary velocity at the inlet of the graft, and being *μ* = 3.2cP the blood viscosity, the shear stress at time t = 0 was recorded to be 6.4 Pa and considered to be an arterial value. Finally *α*_1_ and *α*_2_ are the constant parameters that regulate the cellular events responsible for the hyperplasia, namely cell proliferation, here intended as an average between mitosis and apoptosis, and ECM synthesis. Critical in the coupling of the DS with the CN will be to replace the constant character of *α*_1_ and *α*_2_ in favor of a time dependent trend derived from the gene dynamic.

#### 2. Cluster network (CN)

With our rabbit model, we explored the complexity of shear-mediated vein graft remodeling through a transcriptional profiling. Using a rabbit-specific microarray probe [38], we examined the temporal variation in gene expression within the vein graft wall at 2 hours, 1, 3, 7, 14 and 28 days following the original implantation. The dynamic of all the genes analyzed is reported in the supporting information file S1 Data. We used our customized statistical algorithm [22] in order to organize the genes in 29 different clusters of expression. Using an analysis of variance (p<0.05), minimum effect size (>0.5) and minimum fold change (>0.5) criteria, 13 clusters out of the original 29 were found to have a pattern significantly different once exposed to a different flow condition and for this reason the remaining 16 have not been considered as significant for the purposes of our analysis. Cell proliferation and matrix dynamic are recognized to be the cellular events that mainly drive the hyperplasia. Accordingly, by focusing only on the clusters that are highly populated by genes impacting these cellular events, we further reduced the number of significant clusters to five, which are identified as the primary elements that control the accelerated response to low shear conditions. Finally, we used a repository of gene networking information IPA ingenuity [39] to identify the association of the genes belonging to the significant clusters with upstream biologic mediators and with downstream biologic events. While the relation with the upstream mediators will be addressed in future developments, the relation between clusters and biologic event will be one of the keys of the present work.

**Fig 4:**
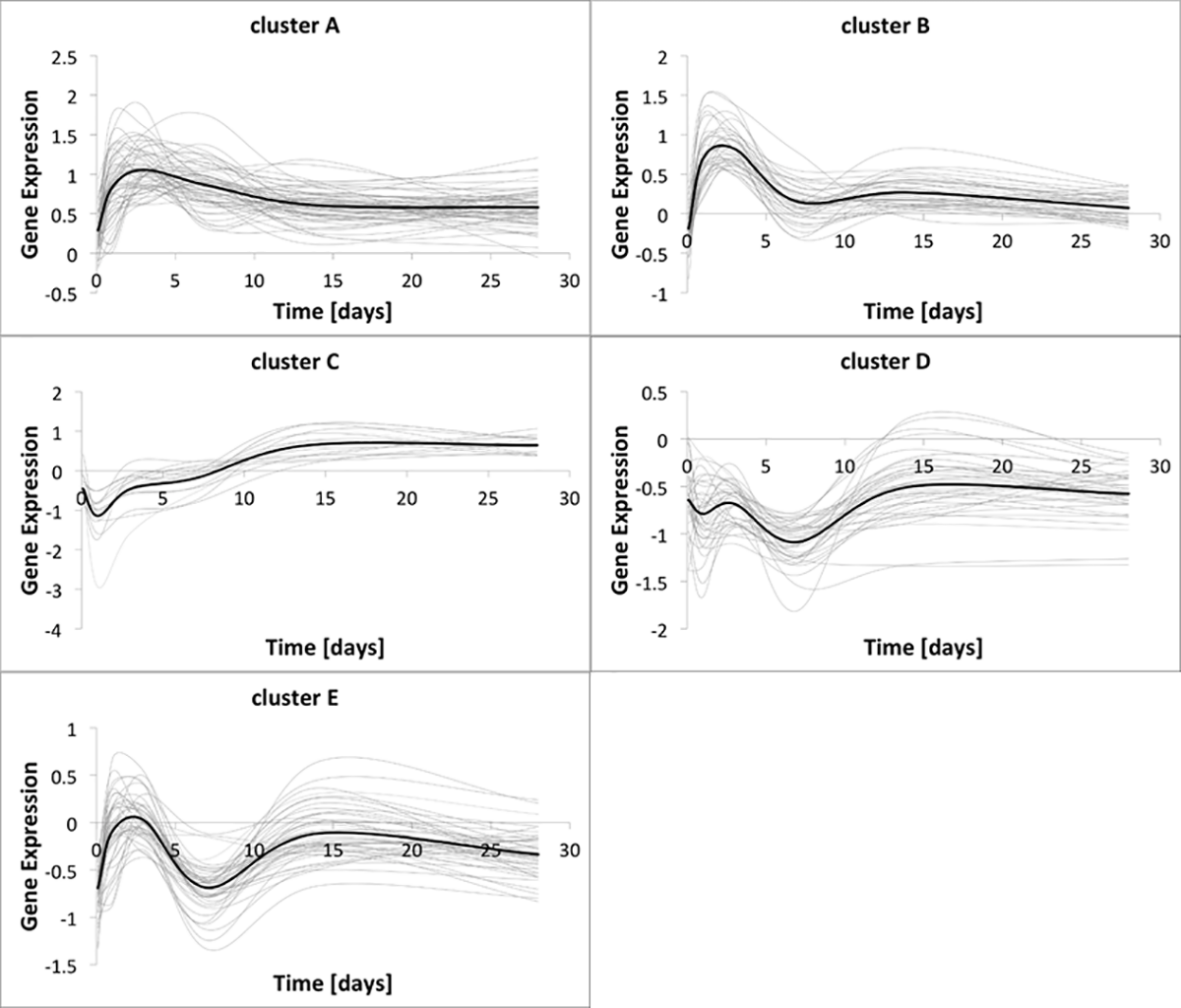
Genes dynamic retrieved from microarray probe for the five significant clusters. In each graph, the dynamic of each gene is represented with a light gray solid line, while the cluster expression, intended as the mean of the genes expression, is represented with a black solid line.

Fig 4 shows the expression of the five significant clusters, labeled from A to E along with the expression of the genes belonging to each specific cluster.

Gene ontology analysis shows a common behavior for the five clusters. An initial response during the first week following implantation corresponds to the inflammatory state that follows the implantation. The excitation lowers starting from Day 7, when the expression starts to stabilize around an asymptote.

We replicated both the expression of each single cluster, and the level of interconnectedness between them. Two main properties of the genes clusters drove our modeling approach: i) their mutual interconnectedness, and ii) their impact on the main cellular events leading the restenosis (i.e. SMCs proliferation and death, and ECM synthesis).

The different clusters have a certain level of mutual interconnectedness that can be expressed by organizing them in a highly integrated network, so that a variation of expression in one single node leads to adaptive changes in the other interconnected components. The choice of a mutual interconnected network allowed us to replicate the property for which a variation of expression in one single cluster influences the level of expression of all the others that are connected to it. This feature is fundamental in order to study the effect of a gene therapy, as it is to be expected that the alteration of one element brings a certain level of activity modifications in other components too, potentially causing secondary effects on the outcome, which must be taking in consideration.

To reduce the problem to one that is sufficiently powered to support a discrete solution, the model will be constructed around gene clusters, which are composed of a set of genes with a unique temporal expression pattern and similar biologic function. Potential interventional strategies can then be contemplated for key elements or upstream regulators within the most influential clusters.

In order to build the skeleton of the CN, we started from a system of Ordinary Differential Equations (ODE), where each cluster’s dynamic is described with one equation, for a total of 5 as the number of clusters identified by the clustering algorithm. The choice of an ODE system is driven by the need of replicate the temporal dynamic of the single cluster along with the level of interconnectedness among the clusters, as previously introduced. Every cluster dynamic is dependent from the dynamic of all the other clusters and it is so mediated through a constant factor A_jk_ that represents the impact carried out by cluster j on cluster k, as it will be described later. The ODE system writes

$$\left\{ \begin{array}{l} \frac{d}{dt}G_{1}=\lambda \left(t\right)\left[\begin{array}{c} A_{11}\left(G_{1}-B_{1}\right)+A_{21}\left(G_{2}-B_{2}\right)+A_{31}\left(G_{3}-B_{3}\right)+A_{41}\left(G_{4}-B_{4}\right)+\\ A_{51}\left(G_{5}-B_{5}\right) \end{array}\right]\\ \frac{d}{dt}G_{2}=\lambda \left(t\right)\left[\begin{array}{c} A_{12}\left(G_{1}-B_{1}\right)+A_{22}\left(G_{2}-B_{2}\right)+A_{32}\left(G_{3}-B_{3}\right)+A_{42}\left(G_{4}-B_{4}\right)+\\ A_{52}\left(G_{5}-B_{5}\right) \end{array}\right]\\ \frac{d}{dt}G_{3}=\lambda \left(t\right)\left[\begin{array}{c} A_{13}\left(G_{1}-B_{1}\right)+A_{23}\left(G_{2}-B_{2}\right)+A_{33}\left(G_{3}-B_{3}\right)+A_{43}\left(G_{4}-B_{4}\right)+\\ A_{53}\left(G_{5}-B_{5}\right) \end{array}\right]\\ \frac{d}{dt}G_{4}=\lambda \left(t\right)\left[\begin{array}{c} A_{14}\left(G_{1}-B_{1}\right)+A_{24}\left(G_{2}-B_{2}\right)+A_{34}\left(G_{3}-B_{3}\right)+A_{44}\left(G_{4}-B_{4}\right)+\\ A_{54}\left(G_{5}-B_{5}\right) \end{array}\right]\\ \frac{d}{dt}G_{5}=\lambda \left(t\right)\left[\begin{array}{c} A_{15}\left(G_{1}-B_{1}\right)+A_{25}\left(G_{2}-B_{2}\right)+A_{35}\left(G_{3}-B_{3}\right)+A_{45}\left(G_{4}-B_{4}\right)+\\ A_{55}\left(G_{5}-B_{5}\right) \end{array}\right] \end{array}\right.$$ (4)

where G_i_ = 1,…,5 represents the expression of the i-th cluster, while B_i_ = 1, …, 5 is the parameter driving the asymptotic trend of the i-th cluster. *λ*(*t*) is a third order polynomial function that serves as time modulation used to drive the dynamic of the cluster expression toward its reference trend described by the experimental data. The output of the basic model expressed with (4) is indeed a linear combination of exponential functions, and the solution of it can either converge to an asymptote, diverge toward infinite, or result in high frequency oscillations trend. On the other hand, from the analysis of the experimental data shown in Fig 4, we observed a common trend for the dynamic of all the clusters, which share a non-monotonic dynamic characterized by one inflection point that perfectly mimics the inflammatory phase the vein faces in the early post-surgical follow-up, and a final asymptotic trend that mimics the post-inflammation relaxation. To be able to catch the non-linearity of the clusters’ dynamic, we applied a modulation mask described by the following:

$$\lambda (t)=C_{1}t^{3}+C_{2}t^{2}+C_{3}t+1$$ (5)

Finally A_jk_, j = 1,…,5; k = 1,…,5 describes the level of incidence that the j-th cluster carries out on the k-th cluster. It is necessary to highlight how A_jk_, B_i_ and C_n_ (n = 1,2,3) are unknown parameters that have to be retrieved from experimental data. Among the unknowns, A_jk_ certainly carries the most valuable information. Indeed, starting from it, we can define a matrix associated to the cluster network, which precisely defines the level of mutual incidence between the various clusters (S1 Table). Basing on this matrix, a network that respects the different level of interconnectedness between clusters can be defined.

Using curated ontology information, genes can be linked to specific biologic processes, i.e. genes belonging to a specific cluster can impact one or more cellular activities. With respect to vein graft adaptation, we have already mentioned in the introduction how the leading cellular events are cell proliferation and death, and ECM synthesis. Within the construct of the clustering model, individual gene sets in each cluster can be mapped to each of the three processes, and these subsets of clustered genes can be assumed to form an integrated network by themselves. Consequently, we deal now with three different network of clusters, one per cellular activity. Each of these distinct networks will be labeled CN1, CN2, and CN3, respectively mapping to the biologic activities of cell mitosis, cell apoptosis and ECM deposition.

In each network, the pattern of expression for each cluster is known from experimental data and this allowed us to retrieve the precise level of interconnectedness among clusters belonging to the same network (A_jk_), also along with the other unknown variables (B_i_, and C_n_). For each network, to retrieve the unknown parameters is equivalent to calibrate the general mathematical model described with (4) on the correspondent experimental data, that are different between SMCs proliferation and death, and ECM synthesis. Indeed, generally speaking, the calibration of a model is the task of adjusting an already existing model to a reference system, typically by minimizing an objective function defined *ad hoc*. In our case, we want to adjust the general cluster network to each experimental dataset by retrieving the value of the unknowns. S1 Fig shows the conceptual diagram followed in order to calibrate the model and to retrieve the unknowns. It corresponds to the red dashed box already seen in Fig 2 and now further described step-by-step in S1 Fig. The calibration was performed by minimizing the distance between the output of the general cluster network, which is parameterized in A_jk_, B_i_ and C_n_, and the correspondent experimental data. The distance is evaluated using the Root Mean Square (RMS) deviation, which is also function of A_jk_, B_i_ and C_n_, and that describes the objective function that has to be minimized. It writes

$$RMS=\sqrt{\sum_{r=1}^{N}\sum_{t=1}^{M}{\left(x_{rt}^{Ref}-x_{rt}^{Mod}\right)}^{2}}=f(A_{jk},B_{i},C_{n})$$ (6)

N = 5 is the number of clusters belonging to the network, while M = 6 is the number of time points at which the vein graft has been harvested. $x_{rt}^{Ref}$ is the expression of the r-th cluster at the t-th time point retrieved from the experimental data (reference of the calibration), while $x_{rt}^{Mod}$ still stands for the expression of the r-th cluster at the t-th time point, but referred to the general network model to be fitted. The objective function has been minimized using a Genetic Algorithm (GA) from the Matlab^®^ Optimization Toolbox. A GA is a method for solving optimization problems based on a natural selection process that mimics the biological evolution [40]. The algorithm repeatedly modifies an initial population of individuals (randomly selected within a pre-defined range), each of them representing a potential solution, by promoting the best and discarding the worst. The right setup of the GA is the key to handle all the potential issues that may occur during the minimization of the objective function. For each network, the high number of unknowns (33), arranges that the algorithm was prone to become stuck in a local minimum instead of a global one. In order to cope with it, the population size of the GA was increased from its Matlab^®^ default value of 50 to 100 time the number of unknowns. Even though in this way the algorithm can explore a wider range of solutions, a too wide initial range can certainly affect the accuracy of the minimization. This issue can be resolved by running the GA recursively, i.e. running the same algorithm several times and by setting as initial range of the n-th run an interval defined inside the proximal surrounding of the solution of the (n-1)-th run. In this specific case we refined in first approximation our solution by running the minimization twice where the initial guess for the second simulation is taken inside the surrounding of the first one. Finally, a penalty factor was added to the objective function in order to maintain the system stable even upon manipulation of the cluster expression that is, after all, the spirit of a potential gene therapy. Specifically, within the definition of the objective function, the best fitting is not only evaluated with the whole group of clusters active, but also with one cluster turn down at each time. For every single evaluation, a threshold tolerance has been set to be Tol = 10 which was believed to be a number sufficient high to provide a reasonable first selection. If the single fitting value has exceeded the tolerance, the current solution would have been discarded *a priori*. In fact, a loss of stability has been observed a posteriori upon manipulation of the network, and in particular by singularly knocking down the expression of most of the cluster. During the minimization process, we systematically tested the best solution found by the algorithm at each step in case of singularly silencing of each single cluster belonging to the network. If the system remains stable knocking the cluster down, the current solution is kept and the algorithm can proceed to the next generation, otherwise the solution is discarded and the algorithm picks another best.

The calibration of the general model on the three series of experimental data allowed us to associate to each biologic event a matrix like the one described with S1 Table and a network reflecting the matrix itself.

#### 3. Sub-models coupling

**Fig 5:**
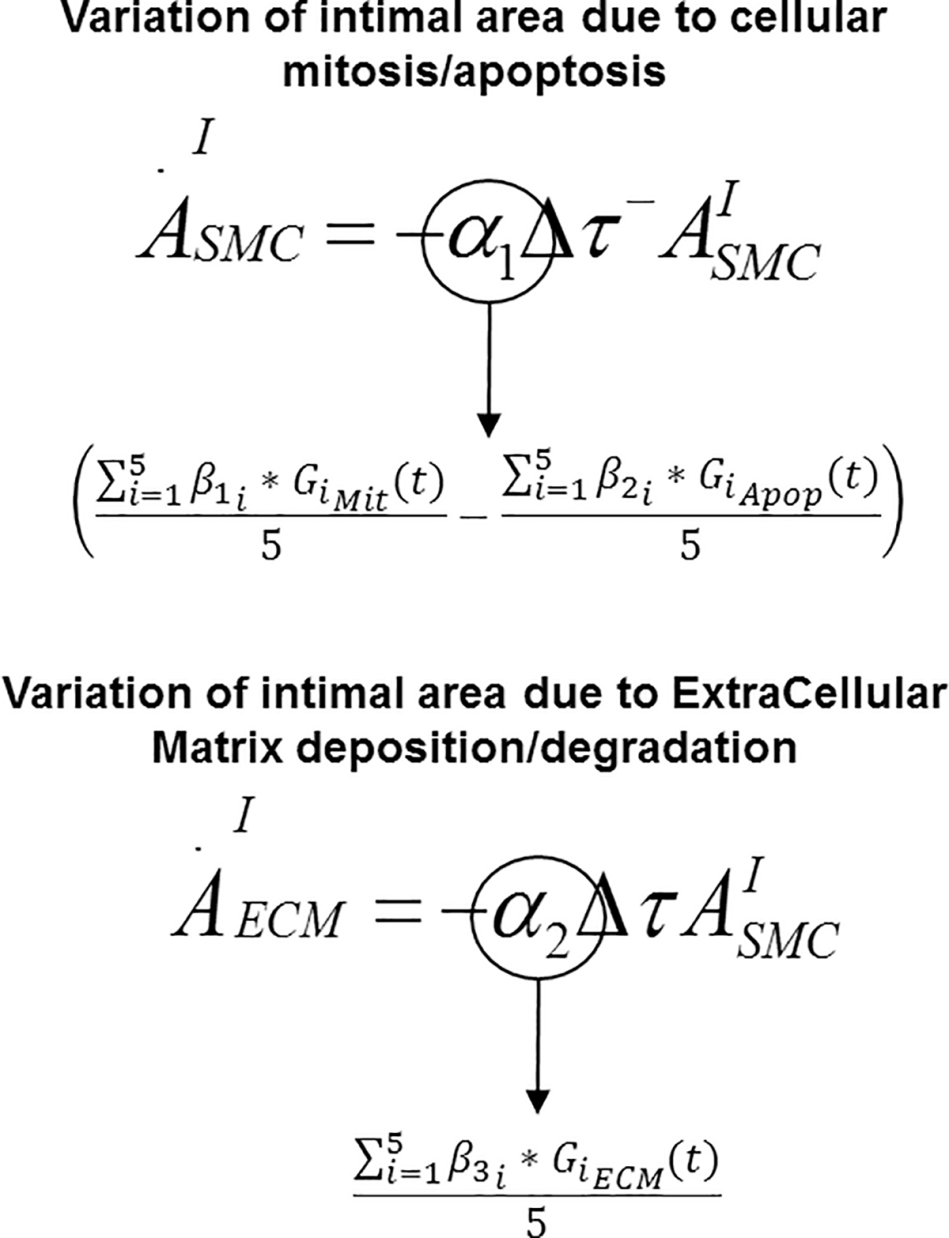
Sub-models coupling. Constant parameters *α*_1_ and *α*_2_ are replaced by the time dependent cluster dynamics mapped to the correspondent biologic event.

The hybrid model was obtained by linking the DS and the three CNs, and specifically by replacing the constant parameter *α*_1_ and *α*_2_ of the DS with the time-dependent cluster expression derived from the CNs and associated to the same cellular event, as shown in Fig 5.

By linking the two sub-models, we detailed the genetic impact on the biologic events that lead the hyperplasia. Indeed the CNs link the dynamic of clusters to the relative activity of the biologic process, while through the DS, the aggregate change in biologic processes can be tracked to predict the net influence on the final vein graft morphology.

In the DS previously introduced, *α*_1_ was designated to control cell proliferation and its constant value was comprehensive of both mitosis and apoptosis. These cell processes are assumed to be directly controlled at the genetic level and accordingly retrieved from their respective CNs, each of them represented by the average of the expressions of the five clusters belonging to the single network. With respect to Fig 5, *α*_1_ is now both cluster and time dependent, and can be written as:

$$\alpha_{1}(t)=\frac{\sum_{i=1}^{N}\beta_{1i}*{G_{i}}_{Mit}(t)}{5}-\frac{\sum_{i=1}^{N}\beta_{2i}*{G_{i}}_{Apop}(t)}{5},$$ (7)

where N is again equal to 5, that is the number of clusters belonging to a single network, ${G_{i}}_{Mit}(t)$ is the time-dependent expression of the i-th cluster belonging to CN1, while ${G_{i}}_{Apop}(t)$ is the time-dependent expression of the i-th cluster belonging to CN2. *β*_1*i*_ is the scaling factor that adapts the unit of measure of the i-th cluster expression belonging to CN1 ([mRNA]) into the hybrid model, and at the same way, *β*_2*i*_ takes care of CN2.

In an analogous manner, *α*_2_ was designated in the DS to dictate ECM deposition kinetics within the wall, and was re-defined via the following expression:

$$\alpha_{2}(t)=\frac{\sum_{i=1}^{N}\beta_{3i}*{G_{i}}_{ECM}(t)}{5}.$$ (8)

Again, ${G_{i}}_{ECM}(t)$ is the time-dependent expression of the i-th cluster belonging to CN3, and *β*_3*i*_ is the relative scaling factor for the i-th cluster into the hybrid model.

The current stage of the hybrid model still represents a basic structure characterized by unknown parameters, such as *β*_1*i*_, *β*_2*i*_, and *β*_3*i*_, for a total of 15 unknowns. These latter are retrieved by calibrating the hybrid model on experimental cell mitosis, cell apoptosis, and ECM deposition data obtained from our rabbit model [41].

#### 4. Clusters weights

Within the same network, each cluster has a different impact on the biologic process to which it is mapped. In order to determine how each network influences its respective cellular event, the relative weight that each cluster employs on the relative cellular event must be defined. This also means that it is necessary to re-visit the definition of *α*_1_(*t*) and *α*_2_(*t*), originally introduced with the DS. Assuming each cluster within a network has a different relative impact on a cellular event, *α*_1_(*t*) and *α*_2_(*t*) can be defined as:

$$\alpha_{1}(t)=\frac{\sum_{i=1}^{N}\beta_{1i}*w_{1i}*{G_{i}}_{Mit}(t)}{5}-\frac{\sum_{i=1}^{N}\beta_{2i}*{{w_{2i}*G}_{i}}_{Apop}(t)}{5}$$ (9)

and

$$\alpha_{2}(t)=\frac{\sum_{i=1}^{N}\beta_{3i}*w_{3i}*{G_{i}}_{ECM}(t)}{5}.$$ (10)

**Fig 6:**
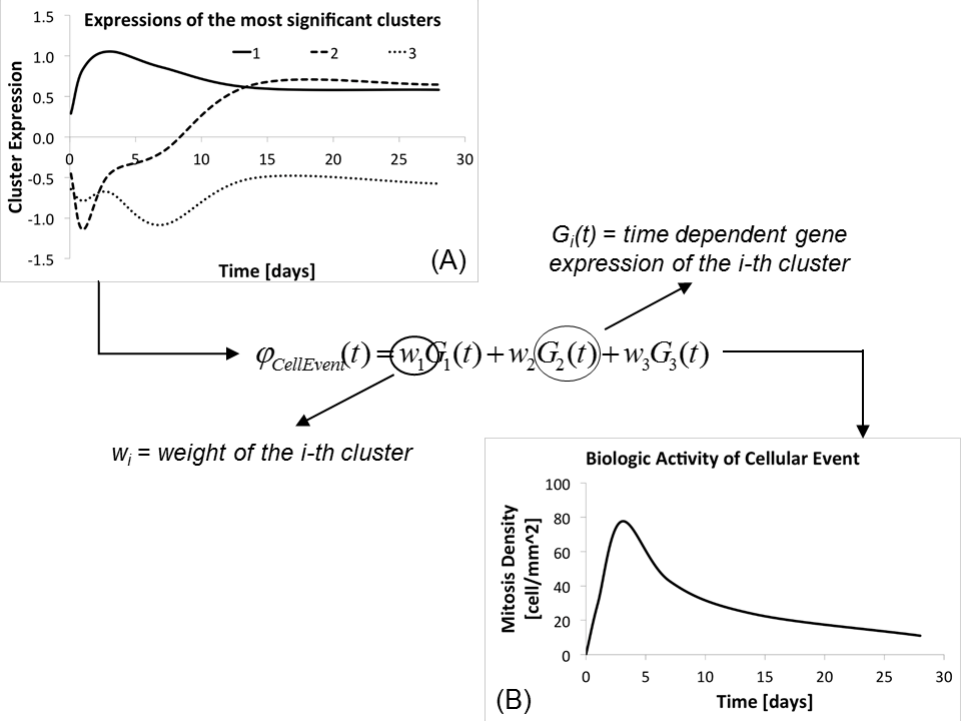
Calibration of the clusters’ weights. The temporal dynamic of a generic cellular event (B) is described with a linear combination of the clusters of expression ontologically related to it (A). The level of impact that a generic cluster (G_i_(t)) employs on the cellular event (*φ*(t)) is mediated through its relative weight (w_i_).

Within these expressions, three new sets of variables have been introduced: *w*_1*i*_ is the weight of the i-th cluster belonging to C1 carried out on cell mitosis, *w*_2*i*_ the weight of the i-th cluster belonging to C2 carried out on cell apoptosis, and finally *w*_3*i*_ the weight of the i-th cluster belonging to C3 carried out on ECM synthesis. *w*_1*i*_, *w*_2*i*_, and *w*_3*i*_ are unknowns and their values were retrieved following the general principle described in Fig 6, which shows a simplified three-cluster version of the calibration. The weighting of the calibration is based on the concept of representing the dynamic of the biologic process (Fig 6B), which is known from experimental data, through a linear combination of clusters’ dynamics (Fig 6A), which are mediated by the different weights that the clusters have on the cellular event.

**Fig 7:**
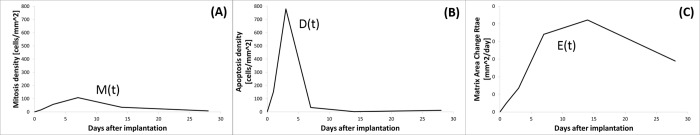
Cellular events dynamic from rabbit model. Cell mitosis rate (A), cell apoptosis rate (B) and ECM synthesis rate (C) are recorded on a post-surgical follow-up of 28 days and harvested at time of implant, and after 2 hours, 1, 3, 7, 14, 28 days.

The temporal evolutions of the three cellular events were retrieved from our rabbit model previously described. For each event, a characteristic variable was recorded at time 0 and after 2 hours, 1, 3, 7, 14, and 28 days from the implant. Fig 7 shows the temporal dynamic of the three cellular events as also reported in the supporting information file S3 Data. Cell mitosis and apoptosis were studied by measuring the SMC concentration within the cross section of the graft as shown respectively in Fig 7A and 7B and reported respectively in S3 Data Proliferation Rate and S3 Data Apoptosis Rate, while the ECM dynamic was studied by measuring the rate of change of ECM area within the graft cross section as shown in Fig 7C and reported in S3 Data Matrix Growth Rate. The temporal dynamic of the cellular events served as reference for the calibration of the clusters’ weights and they were labeled as M(t), D(t), and E(t) respectively for cell mitosis, cell apoptosis, and finally ECM synthesis.

As done for the calibration of the CNs we used a GA in order to minimize an objective function that describes the distance between experimental data and parameterized model, retrieving in this way the unknown weights. The steps followed for the calibration of each set of weights is described in S2 Fig and it reflects the general principle reported in Fig 2 (dashed green box), where the reference is the temporal dynamic of the cellular event, known for experimental data, and the model to be fitted is the linear combination of clusters dynamics mediated by the unknown weights.

Going from a general example to our precise case, the three linear combinations, one per cellular event, were defined as follows:

$$\varphi_{Mit}(t)=\sum_{i=1}^{N}w_{1i}*G_{1i}(t)$$ (11)

$$\varphi_{Apop}(t)=\sum_{i=1}^{N}w_{2i}*G_{2i}(t)$$ (12)

$$\varphi_{ECM}(t)=\sum_{i=1}^{N}w_{3i}*G_{3i}(t)$$ (13)

and their correspondent objective functions write:

$$RMS_{Mit}=\sqrt{\sum_{i=1}^{M}\left({\varphi_{Mit}}_{i}(t)-M_{i}(t)\right)}=f(w_{1i})$$ (14)

$$RMS_{Apop}=\sqrt{\sum_{i=1}^{M}\left({\varphi_{Apop}}_{i}(t)-A_{i}(t)\right)}=f(w_{2i})$$ (15)

$$RMS_{ECM}=\sqrt{\sum_{i=1}^{M}\left({\varphi_{ECM}}_{i}(t)-E_{i}(t)\right)}=f(w_{3i})$$ (16)

Thanks to the limited number of unknowns for each minimization (5), the Matlab^®^ default setup for the GA was already appropriate to reach a reasonable objective function minimization. In addition, two constraints, described with (17), have been applied to the GA: in each network, the value of a cluster weight is included in the interval [-1;1], and the sum of the absolute value of the weights does not exceed a unitary value. The set of constraints writes:

$$\left\{ \begin{array}{c} w_{i}\in [-1;1]\\ \sum_{i=1}^{5}\left|w_{i}\right|=1 \end{array}\right.$$ (17)

With these constraints, we allowed a cluster to affect a biologic process both positively and negatively, founded on the concept that a gene can either enhance or inhibit a defined cellular event.

#### 5. Hybrid model calibration

The hybrid model was calibrated on experimental data accurately described in S2 Data in order to retrieve the unknowns previously introduced (*β*_1*i*_, *β*_2*i*_ and *β*_3*i*_), which values is reported in S7 Table. The calibration scheme is illustrated in S3 Fig following the principle of Fig 2 (blue dashed box). We chose as reference the temporal dynamic of intimal thickness, which was recorded at the harvesting of the grafts from our rabbit model after 2 hours, 1, 3, 7, 14, and 28 days from the original implant as fully described in the supporting information file S2 Data. On the other hand, the basic model is represented by the hybrid model parameterized in *β*_1*i*_, *β*_2*i*_, and *β*_3*i*_. The objective function was defined as the RMS between parameterized model and experimental data as follows:

$$RMS=\sqrt{\sum_{i=1}^{M}\left(I_{i}^{Ref}-I_{i}^{Mod}\right)}=f(\beta_{1i},\beta_{2i},\beta_{3i})$$ (18)

$I_{i}^{Ref}$ is the temporal dynamic of the wall thickness recorded from the experimental data, while $I_{i}^{Mod}$ is the intimal thickness dynamic as output of the hybrid model parameterized in *β*_1*i*_, *β*_2*i*_, *β*_3*i*_. M = 6 is the number of time points in correspondence of which the grafts were harvested and the intimal thickness was recorded, and i = 1,…,5 identifies again the single cluster inside the network. The goodness of the calibration was evaluated both qualitatively, by plotting in the same graphic both the experimental evidences and the hybrid model output, and quantitatively through the Percentile Root Mean Square (PRMS) deviation calculated between reference and hybrid model:

$$PRMS=\sqrt{\sum_{j=1}^{M}\left(I_{i}^{Ref}-I_{i}^{Mod}\right)}*100$$ (19)

Finally, after having integrated the DS with the networks dynamic, i.e. after having replaced (9) and (10) in (2), the hybrid model assumes its final form fully described by:

$$\left\{ \begin{array}{c} {\dot{A}}_{SMC}=-[\frac{\sum_{i=1}^{5}\beta_{1i}w_{1i}\;{G_{i}}_{Mit}(t)}{5}-\frac{\sum_{i=1}^{5}\beta_{2i}{{w_{2i}\;G}_{i}}_{Apop}(t)}{5}]\;\Delta \tau^{-}A_{SMC}\\ {\dot{A}}_{ECM}=-[\frac{\sum_{i=1}^{5}\beta_{3i}w_{3i}\;{G_{i}}_{ECM}(t)}{5}]\;\Delta \tau^{-}A_{SMC},\;if\;A_{ECM}>0,\;and\;0\;otherwise \end{array}\right.$$ (20)

where all the variables have been already fully described in the previous sections.

### *In silico* gene therapy

#### 1. Gene therapy as a tool to minimize hyperplastic growth of the wall

**Fig 8:**
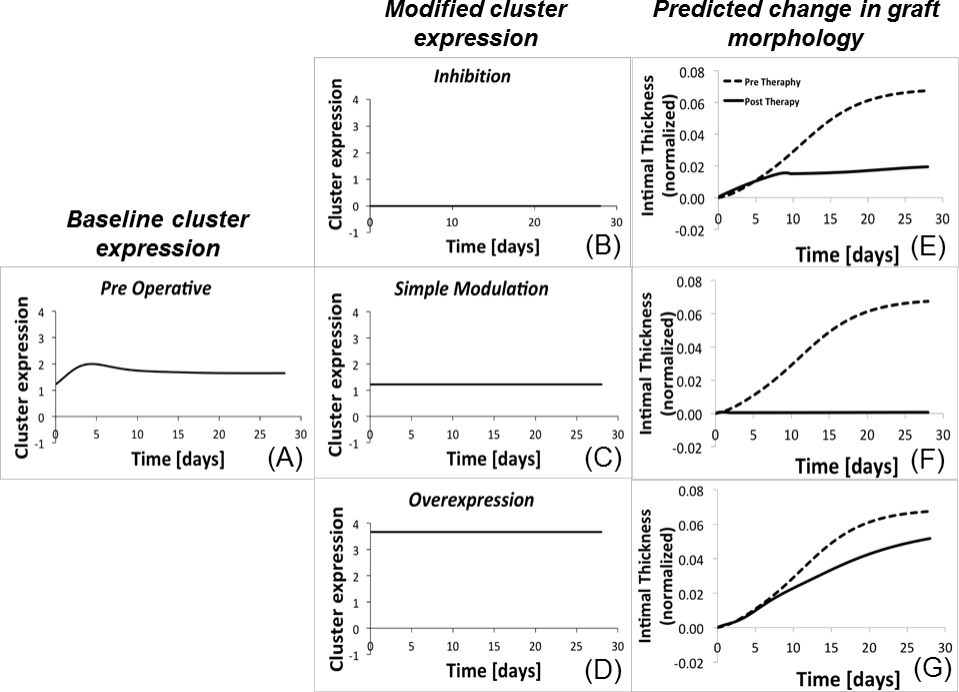
Gene therapy general concept. With the current analysis, a cluster dynamic (A), is modulated from its initial condition in three distinct ways: complete inhibition of cluster expression (B), freezing cluster expression at the initial (baseline) condition (C), and fixed overexpression of the cluster expression (D). The modification in cluster expression alters the cell or ECM kinetics, leading a new trajectory for hyperplastic growth of the wall (E)-(G). Dashed line represents the dynamic in absence of therapy and the solid line, the dynamic post-therapy.

In general, our hybrid model offers us the possibility to modify the profile of clusters expression to generate an impact on the vein graft morphology. The primary goal is to identify a potential modifier of gene expression (termed gene therapy) that optimally alters the cluster expression such that there is a reduction in wall cross-sectional area at one month following implantation. A theoretical example of these potential scenarios is outlined in Fig 8.

Starting from Fig 8A, which shows the expression of a theoretical cluster in the absence of any genomic manipulation, we defined as virtual gene therapy any kind of alteration of the original expression that can alter the trajectory of the hyperplastic growth of the wall. We examined three different kinds of perturbation:

- Inhibition (example in Fig 8B), for which the initial level of cluster expression is reduce (to a minimum value of zero, which corresponds to complete silencing) and maintained constant for the duration of the simulation;
- Simple modulation (Fig 8C), where the initial level of cluster expression is fixed at its initial value;
- Overexpression (Fig 8D), that enhanced the cluster expression is enhanced up to 3-fold greater than its initial value and maintained constant for the duration of the simulation.

The common feature to the three different approaches is that once perturbed the initial condition, the cluster expression is maintained to a constant level for the entire post-surgical period. Accordingly, a potential gene therapy of this kind would be implemented in the clinical ambit by continuously administering to the patient a drug able to alter the level of targeted gene expressions and to maintain it constant for the entire follow-up. This is of course only a tentative choice. More therapies implementations will be explored through future developments of the model. Fig 8E–8G illustrate the potential trajectory of the hyperplastic response that results from the change in cell and ECM kinetics associated with each virtual gene therapy.

**Fig 9:**
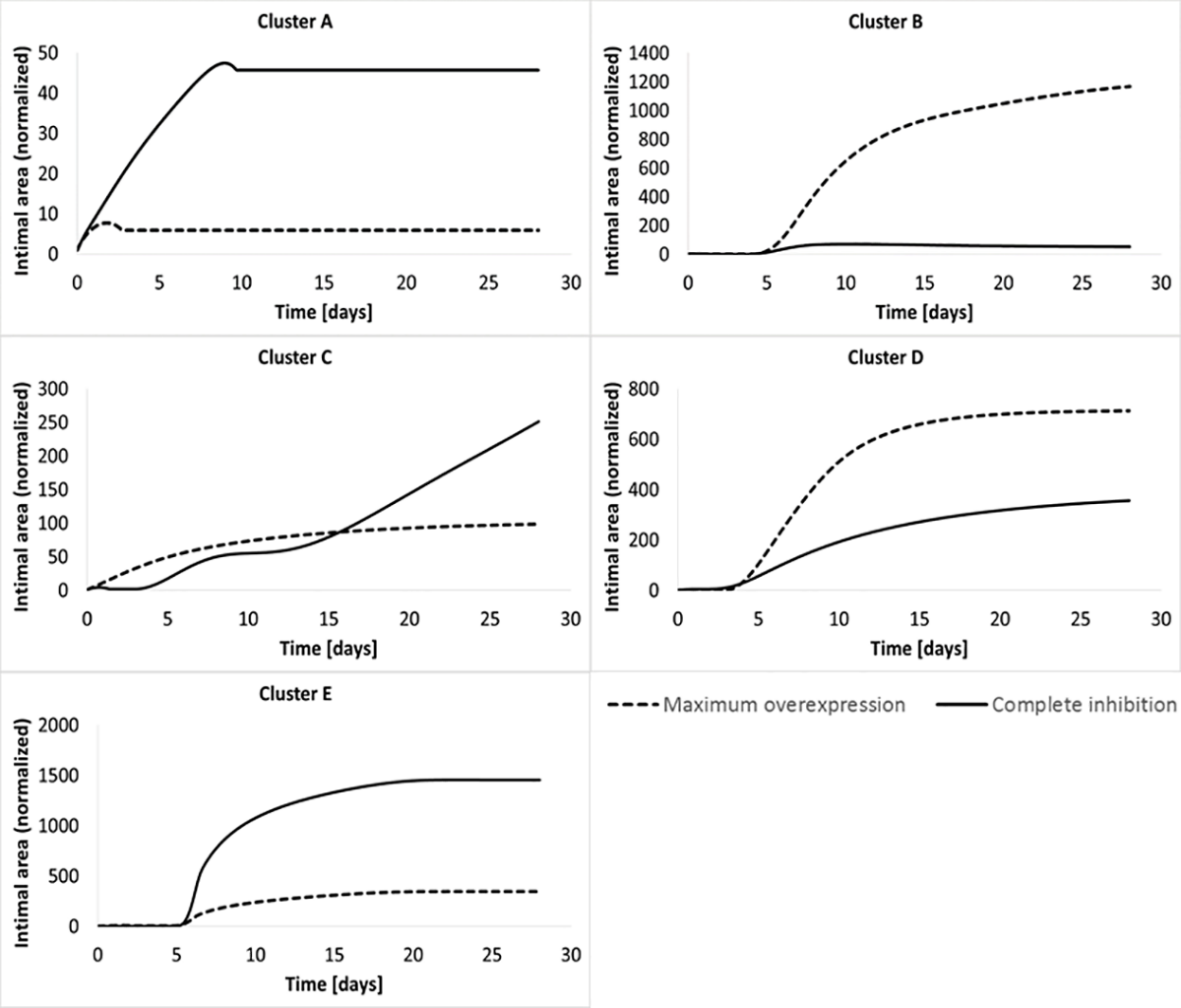
Gene therapy principle. Comparison between thicknesses of the wall dynamic altered with cluster set at maximum overexpression (dashed line) and complete inhibition (solid line).

We simulated computationally the gene therapy by directly acting on the CNs, which general form was described in (4). Following the concept illustrated in Fig 8, we examined various genomic manipulations using the following strategy:

1. *Gene therapy model*: We applied a specified perturbation on the initial condition of the cluster dynamic maintaining then the level of expression constant for the entire follow-up. Assuming a collapsed form of (4), the therapy principle translates into the following:
  $$\left\{ \begin{array}{c} \frac{\partial }{\partial t}G_{i}(t)=0\\ G_{i}(t=0)=\delta_{i}*G_{i}(t=0) \end{array}\right.$$ (21)
  As already seen in (4), *G*_*i*_(*t*) stands for the expression of the i-th cluster. The set of constants *δ*_*i*_ defines the entity of the perturbation applied to the initial cluster dynamic, which remains then constant as the variation of cluster expression is null. Furthermore, *δ* < 1 indicates a reduction of cluster activity, *δ* > 1 an increase of cluster activity, and finally *δ* = 1 a cluster activity unvaried.
2. *Single cluster modulation*: we initially studied the minimization of thickness of the wall in case of single cluster alteration, where each cluster has been modified singularly, leaving all the others unvaried (a total of five gene therapies simulated, one per cluster). Fig 9 provides a confirmation of the extreme heterogeneity and of the lack of linearity of gene therapy outcomes.
  The figure shows, for each single cluster, the morphology outcome in case of constant modulation both with complete inhibition (solid line) and maximum overexpression, set at 3-fold over baseline (dashed line). Keeping in mind that the golden standard is to minimize as much as possible the thickness of the graft wall, it is clear how some clusters might provide good therapeutic potential if over-expressed at their maximum value, like cluster B and D, while some others might be efficient if modulated to their lowest value, like cluster A and cluster E. Also the intrinsic outcome variation among different clusters has to be taken in consideration. It is clear how some clusters bring with them a wider range of outcome variation than others, like cluster B compared with cluster A. The impact of the clusters’ modulation has been evaluated by implementing a Matlab^®^ code *ad hoc* and the same is valid for the coupled clusters modulation about to be introduced.
3. *Coupled clusters modulation*: Stemming from the concept that there is significant redundancy within the interconnected gene network and multiple targets might be required to achieve notable improvements in the outcome morphology, an analysis involving the simultaneous manipulation of two clusters was performed. Two clusters were modulate within the same range described for the single modulation at the start of the simulation, leaving the other 3 unvaried, leading to the investigation of 10 new gene therapies.
4. *Evaluation of the outcome*: In order to evaluate the efficiency of each gene therapy, we focused both on a visual evaluation comparing the pre-therapy and the post-therapy trend, but mostly on a numerical evaluation of the intimal area at the end of the post-surgical follow-up comparing pre-therapy and post-therapy. With the goal of reducing the hyperplastic response of the wall to minimize narrowing of the graft lumen, success of a gene therapy was defined as the normalized difference in cross-sectional area of the graft at the completion of the simulation, given by the following expression:
  $$gain\%=\frac{A_{i}^{pre}-A_{i}^{post}}{A_{i}^{pre}}*100,$$ (22)
  where $A_{i}^{pre}$ is the area of the intima recorded after 28 days of follow-up in pre-therapy conditions, while $A_{i}^{post}$ represents the same morphologic variable, recorded at the same time, but post-therapy. To constrain the path of the remodeling to biologically plausible solutions, a penalty factor was added to the GA in order to maintain cell mitosis and apoptosis must remained positive throughout the 28-day simulation. Solution not conforming to this standard were discarded and the algorithm reinitiated at the last viable solution. Simulating the outcome of a single cluster intervention required an approximately 10 minutes using an Intel(R) Xeon(R) CPU E3-1270 V2 @ 3.50GHz machine run in parallel computing regime.

The choice of altering the clusters dynamic either singularly or in a pairwise way is corroborated by the need of maintaining a balance between therapy effectiveness and biological feasibility. The overall goal is indeed to reach the best impact on the pathology with a post-surgical therapy that does not massively revolutionize the subject’s genome. Indeed, by augmenting the number of gene modifications, we also run the risk of increasing the number of undesired and uncontrollable side effects that may also lead to different gene-based pathologies.

#### 2. Sensitivity analysis

A minimization algorithm, such a GA, is similar to dynamics of peptide folding, where a protein in its secondary structure will randomly and stochastically explore different conformations in order to reach a minimum energy state [42]. Analogous to a folded protein being locked in a local minimum energy state is the potential that the GA identifies a local minimum as the optimum solution. A sensitivity analysis to explore the parameter space around the solution is an effective approach to test the validity of the minimum solution. As such, for each optimum single- and dual-cluster solutions (*δ*_*min*_, defined by the fixed cluster activity between zero and 3-fold that yielded the maximum reduction in wall area) a sensitivity analysis was conducted. Solutions were examine through a 50% parameter space.

## Results and discussion

In our formulation, we extensively performed non-linear fittings in order to validate our multiscale model on experimental data. The validation encompassed various scales, from gene level to tissue level passing through cellular level. The common denominator has always been the extensive usage of GAs as described in the correspondent Methods section. For every calibration process, we applied the principle described by Mitchell M [43] in order to ensure the robustness of the convergence. In our case, this translated into running the GA multiple times and in choosing among the different solutions the one that carried with it the least percentile error. A further improvement will consist in the usage of a metaheuristic approach as described by Gendrau M [44]. Accordingly, a more robust solution will be achieved by combining a global minimization algorithm (the GA already used) with a local one, like a Simplex based method.

### 1. Clusters interconnectedness

**Fig 10:**
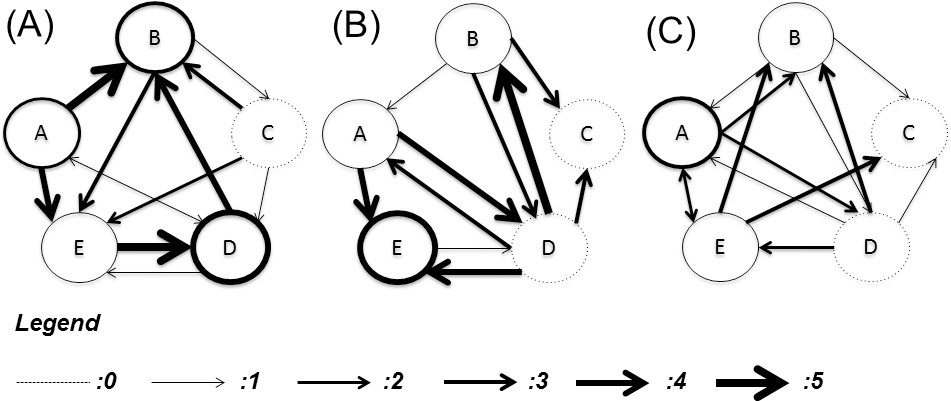
Interconnected networks of clusters. Networks mapped to cell mitosis (A), cell apoptosis (B), and ECM synthesis (C). For illustration purposes the level of interconnectedness between networks was divided in a range from 0 (dashed line for the cluster self-enhancement and no line for different clusters interconnectedness representing no impact) to 5 (thick solid line representing the maximum impact).

The general model of CN, defined by Eq (3), was calibrated on the experimental data obtained by simultaneously with the genomic data and describes for cell mitosis (obtaining CN1), cell apoptosis (CN2), and ECM synthesis (CN3). For each network, by minimizing the associated objective function [Eq (5)], we retrieved the unknowns of the system which also corresponds to define the matrix A_jk_ associated to the specific network. The specific values of A_jk_ for CN1, CN2, and CN3 are respectively reported in S2 Table, S3 Table and S4 Table. Also the values of B_i_ and C_n_ are reported for all the cellular events considered respectively in S5 Table and S6 Table. As the network interconnectedness is assumed to be unique for each of the three biologic events, we retrieved an independent solution for each of the three matrices. The network maps for mitosis, apoptosis, and ECM synthesis are shown in Fig 10.

Notable variability in the relationships among clusters is evident, with some clusters demonstrating a strong impact within one network while these the same clusters show little to no connectedness within a different network. Not surprisingly, other clusters maintain their mutual influence across all the networks, as was noted with cluster D-B interaction where cluster B maintains a maximum influence on cluster B independent of the biologic function. It is important to note that the relationships among clusters was assumed to be independent of time, to enforce the concept that the biologic pathways that define interconnectedness are fixed by the intrinsic biology of the organism.

In order to deal with the potentiality of over fitting, our group is currently exploring some alternative strategies. A first approach consists in using a non-linear stability analysis in order to reduce the number of parameters needed to fit the model on the experimental data. A second approach focuses on one hand on increasing our dataset with low fidelity data that would be easier to recover experimentally. This kind of approach has also been nicely described by Parnassini et al. [45]. On the other hand, we will need to use a more detailed experimental setup for validation purposes, combining for example low, medium, and high shear stress data.

### 2. Clusters weights

**Fig 11:**
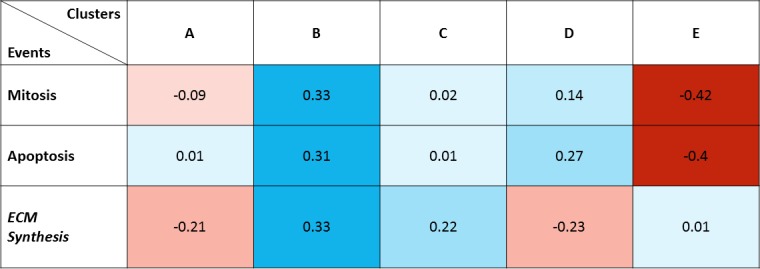
Clusters weights. Relative weights of the five clusters on each of the 3 biological processes. Positive weights are mapped in red and negative weight in blue, with tonality from light (low impact) to dark (high impact).

Critical in our formulation is the linkage between gene cluster interconnectedness and biologic function. As such, each network must be calibrated to the experimental data that describes the time-dependent changes of each biologic event. For each network, we defined a vector $\vec{w}=\{w_{1},w_{2},\ldots ,w_{5}\}$, where *w*_*i*_ represents the relative impact that the i-th cluster has on the biologic event associated to the network (Fig 11).

Note that a cluster can have either a positive or a negative influence on an events, indicating that a cluster can either enhance or prevent that biologic activity. At this stage, interesting patterns that may have important implications for gene therapy opportunities begin to emerge. For example, manipulation of cluster B may be a promising given that it employs a high impact on all the cellular events. More complex manipulations also begin to emerge where the differential patterns of activation can be leveraged. This is seen in the clusters B and E, where cluster B inhibits all events while cluster E inhibits all events except for ECM synthesis, where it has no notable biologic impact. With the goal to reduce wall thickening by inhibiting cell proliferation/ECM synthesis and increasing cell death, Cluster D presents an interesting opportunity. Although its effect on biologic activity is generally less than other biologic clusters, it has a different effect on mitosis/apoptosis and ECM synthesis. Whereas clusters B and E maintain a similar influence across all the biologic processes, cluster D differentially enhances apoptosis greater than mitosis, while negatively impacting the rate of ECM synthesis.

The same considerations reported previously about the potential insurgence of over fitting or about the robustness of the minimization algorithm are still valid for cluster weights too.

### 3. Calibration of the hybrid model

**Fig 12:**
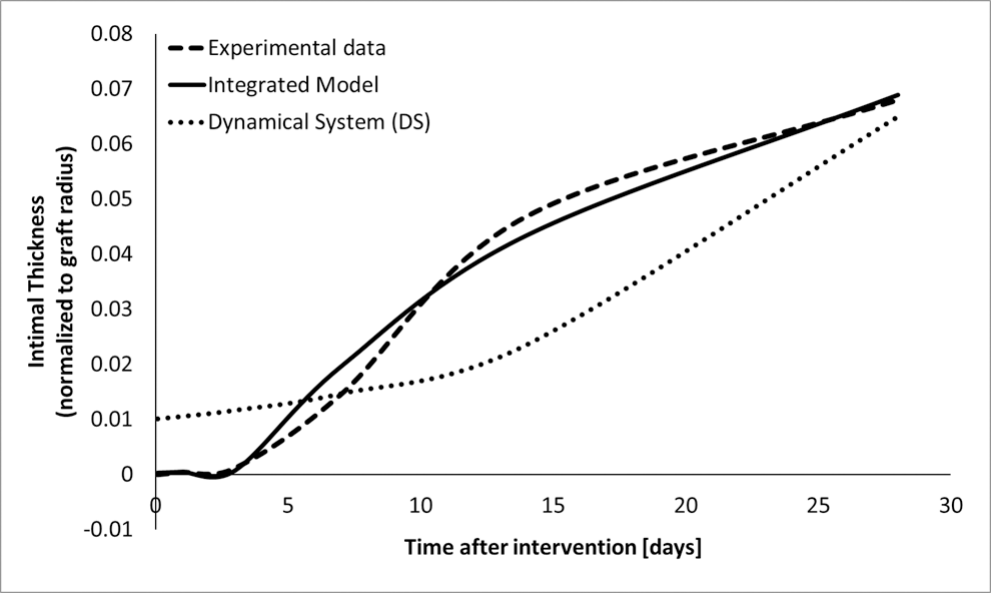
Calibration of the hybrid model. The temporal dynamic of intimal thickness is recorded as output of the DS (dotted line), from experimental data (dashed line), and as output of the integrated model (solid line).

The integration of the DS with the CNs not only links the gene level to the cellular and tissue level, but also moves the entire model closer to the experimental reality. The result of the hybrid model calibration (Fig 12) illustrates how the exponential growth of the wall thickness, recorded as output of the DS, is only partially representative of the real progression appreciated from the experimental data. By integrating the sub-models, we changed the time-invariant character of its driving parameters, providing a mechanism for time-dependence that drives the hybrid model according to the dynamic gene expression pattern.

From a qualitative perspective, we were able to replicate the experimental evidences with a high degree of accuracy. Also, through the evaluation of the PRMS between experimental data and hybrid model, defined with (20), we were able to quantitatively describe the goodness of our approximation, which is confirmed to be very accurate with a PMRS less than 1%. Finally, the over fitting issue has been handled as previously described.

### 4. Gene therapy

We previously described how we simulated several gene therapies by directly minimizing the intimal thickness, that was function of the variable *δ*, representing the level of initial alteration imposed to the cluster dynamic. Using this structure, a therapy was considered effective when the predicted cross-sectional area of the wall following the manipulation was less than baseline (i.e. the non-interventional area) 28 days after graft implantation. We examined both single and coupled cluster modulations, integrating a sensitivity analysis with each prediction in order to test the robustness of the solution.

#### Single cluster modulation

**Fig 13:**
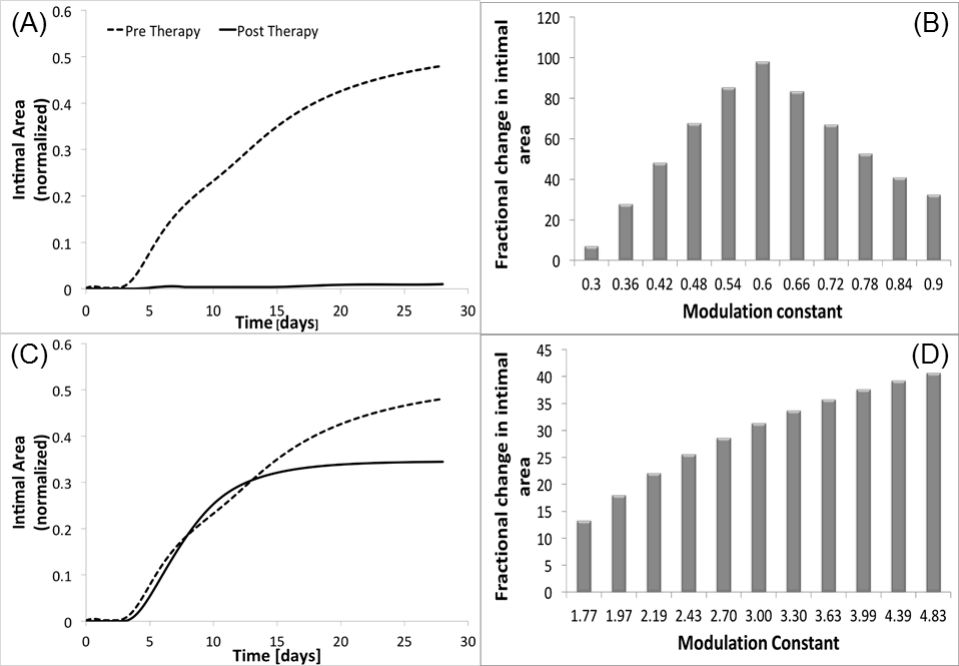
Gene therapy: Single cluster modulation. Reduction of intimal area recorded by singularly modulating cluster C (panel A) and cluster D (panel C). The corresponding sensitivity analyses are clusters C and D provided (panel B and D, respectively).

A single gene therapy cluster modulation was performed, where the optimum magnitude of inhibition or overexpression (*δ*) ranged from zero to 3. Two clusters (C and D) were identified as promising candidates (Fig 13). These quantitative results confirm some of the initial observations from our qualitative analysis of the cluster weighting observations. Cluster C is the most promising, where inhibition to 60% of its initial value (*δ*_*C*_ = 0.6) and fixing the expression at this level resulted in a 98% reduction in wall area at 28 days. Cluster D modulation also resulted in notable improvements, with a 300% augmentation of the initial expression (*δ*_*D*_ = 3) leading to 33% reduction in the 28 day wall area.

The sensitivity analysis for cluster C (Fig 13B) demonstrates a relatively sharp peak around 0.6, with more potent over- or under-expression leading to a sharp reduction in effectiveness. In contrast, the solution for cluster D does not represent a local maximum, but was returned as the optimum solution due to the imposed on maximum over-expression. While this 3-fold limit was enforced to model the physical reality that there is finite increase in gene over-expression that can be achieved, it is admittedly arbitrary and will undoubtedly vary for individual genes or sets of genes, However, from a systems point of view, an unbounded solution may induce marked instability and lead to a final morphology that cannot be achieved in physical reality. Further experimentation and the integration of an expression limit that is tailored to the biology of cluster D would be required to further define the potential therapeutic utility of this cluster.

#### Coupled cluster modulation

Several underlying philosophies guided us in performing dual cluster modulations: i) single target approaches have universally failed as effective clinical therapies, in large part secondary to inherent redundancies in the system; ii) by altering two clusters simultaneously, a wider range of potential therapies (from 5 possibilities to 10) could be explored; and iii) such an approach can leverage cluster-specific differences in their biologic effect, resulting in a potential synergy that cannot be achieved by single cluster modulation.

**Fig 14:**
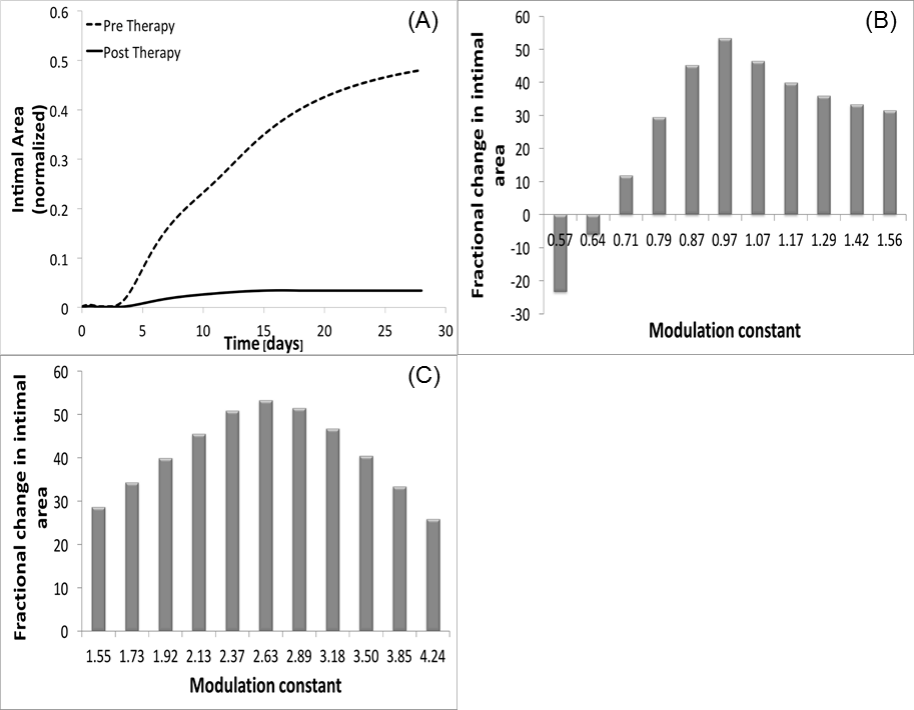
Gene therapy: Coupled cluster modulation. Reduction of intimal thickness recorded by modulating cluster C and D simultaneously (A), with sensitivity analysis associated for cluster C (B) and cluster D (C).

Among the potential emergent behaviors that can be observed with dual-cluster modulation there is an improved stability of the optimum solution. This can be seen with clusters C and D, which individually were identified as the promising solutions. While the dual cluster modulation result paralleled the reduction in wall thickness that was observed with One coupled clusters modulation resulted as promising to reduce the restenosis phenomenon, i.e. the simultaneous modulation of cluster C (through a new constant *δ*_*C*_) and cluster D (through a new constant *δ*_*D*_)), that not surprisingly are the clusters that generated a positive impact if modulated individually. Fig 14A again shows the comparison between the intimal area dynamic recorded in absence of therapy and post therapy. Qualitatively the reduction of intimal area at t = 28 days is evident, something that it is confirmed also quantitatively, indeed we estimated a gain% = 94%, which is a value very close to the single modulation of cluster C case. The therapy performed considers not anymore a single perturbation, but a couple identified by (*δ*_*C*_, *δ*_*D*_), that describes the level of initial perturbation to be applied to cluster C and D respectively. From our analysis, *δ*_*C*_ = 0.97, which means that the initial expression of cluster C has been almost left unvaried, and *δ*_*D*_ = 2.64. Again, both the clusters’ dynamics have been modified at time t = 0 and then constantly expressed during all the follow-up.

An interesting consideration is retrievable from Fig 14B and 14C that show respectively the sensitivity analysis conducted on *δ*_*C*_ and on *δ*_*D*_. Cluster C certainly maintains its robustness as in the single modulation case, but this time even cluster D shows the same property, with a histogram qualitatively very similar to the one appreciated for cluster C in Fig 13B. This confirms our second hypothesis, for which the simultaneous modulation of cluster C and D makes also the perturbation of D as robust as C. However, being the gain% associated with the simultaneous modulation of C and D lower than the one retrieved with the single modulation of C, the most promising gene therapy still consists in halving the initial expression of cluster C maintaining it constant for all the follow-up. In this way we were able both to retrieve the best gain% and to obtain a robust solution.

## Conclusions

The current manuscript presents the architecture for tracking an acute perturbation in a biologic system through a multiscale model that links gene dynamics to cell kinetics, with the overall goal of predicting tissue adaptation. Given the complexity of the genome, the problem is made tractable by clustering temporal changes in gene expression into unique patterns. These cluster elements form the core of an integrated network that serves as the driving force for the response of the biologic system. In the current analysis, we use the clinical scenario of vein graft adaptation as an example problem. Vein when placed in the arterial circulation can develop an aggressive hyperplastic response that narrows the lumen, reduces blood flow, and induces in situ thrombosis [4,7]. Reducing this hyperplastic response has been a long-standing but unrealized goal of biologic researchers in the field. With repeated failure of single target therapies, the redundant response pathways are thought to be a fundamental issue preventing progress towards a solution. Using the current framework, we demonstrate how theoretical genomic manipulations can be introduced into the system to shift the adaptation to a more beneficial phenotype, where the hyperplastic response is mitigated and the risk of thrombosis reduced. Using this approach, we are able to examine a multitude of potential opportunities for intervention via gene over-expression or inhibition, delivered in isolation or combination, and induced early or late in the adaptation process.

In this analysis, we utilize our rabbit vein bypass graft data that was presented previously in our development of an algorithm to clusters gene expression dynamics in response to environmental signals [26]. Emanating from this work was the identification of five gene clusters that differentiated the low flow (i.e. pro-hyperplastic) from high flow (i.e. anti-hyperplastic) response. The current analysis is a direct extension of this work, building on these general associations between gene expression and a beneficial phenotypic outcome to creation of a model that identifies those genes sets most likely be of therapeutic benefit.

Critical in any modeling effort is validation of the results outside to specific operating parameters that were used in its creation. In the current effort, we utilized two distinct phenotypes (high flow/pro-hyperplastic and low flow/anti-hyperplastic) that were defined as success or failure. In reality, the biologic response is a continuous spectrum and integration of this complexity is required. In our formulation, exposing vein bypass grafts to flow conditions intermediate to the two extremes offers the opportunity to examine the full range of adaptive responses. Such experimental work has been initiated and will serve as an important extension and validation of the current analysis. It is anticipated that important non-linearities in the biologic response will emerge and be carefully integrated into the current architecture.

Also, as discussed in the Results section, the minimization algorithms will be improved in order to abate the potential lack of robustness and to reduce the insurgence of over fitting phenomena. For the first aspect, a metaheuristic approach will be followed, consisting in the combination of a global minimization method with a local one. For the second aspect, the key will be to find the right compromise between a reduction in the number of parameters and the increase of the experimental data.

The next step in this our development pipeline will be to engage the biology of these critical gene sets. While the current results are informative, the large size of these clusters (ranging from 30 to 150 genes per cluster) only narrows the field of potential therapeutic candidates. From a biologic standpoint, controlling an entire cluster of genes is not feasible but provides a detailed map of how the adaptation response needs to be modified to achieve the desired effect. We will utilize the known interconnectedness among these critical genes sets to identify a key set of upstream regulatory elements that control their activity. This has been completed in another work by our group, and we have identified a core set of upstream regulators that appear to be cornerstone pieces in this biologic response [41]. Integration of these regulators into the current architecture should reduce this set a handful of targets that can be realistically moved towards further experimentation. While it would be tempting to move directly into in vivo biologic testing, using our rabbit vein graft model, a more conservative approach will be utilized. In vitro, cell culture experimentation, where vascular cells are exposed to realistic flow conditions, offers the ability to precisely manipulate gene activity in a well-controlled, reproducible environment. Given the challenges associated with the biologic redundancy of the vein graft adaptive response, such an experimental approach provides the opportunity to examine combinatorial therapies, with the goal of identifying unrecognized synergies among these therapeutics and moving these pieces toward final in vitro validation.

## Supporting information

S1 Fig Calibration of the Cluster Network (CN). The distance between the mathematical model (replicated with an ODEs system) and the experimental data (from rabbit specific microprobe) is minimized with a GA and the unknowns of the CN are retrieved.
(TIF)

S2 Fig Calibration of the clusters weights. A linear combination of clusters dynamics, mediated with the level of activity that each cluster employs on the cellular event, is fitted on the temporal dynamic of the relative biologic process.
(TIF)

S3 Fig Calibration of the hybrid model. The distance between the intimal thickness dynamic recorded from experimental data and the hybrid model is minimized with a GA and the unknowns of the model are retrieved.
(TIF)

S1 Table Extended matrix A_jk_. Mutual level of interconnectedness among clusters.
(TIF)

S2 Table Extended matrix A_jk_ for Mitosis. (TIF)

S3 Table Extended matrix A_jk_ for Apoptosis. (TIF)

S4 Table Extended matrix A_jk_ for ECM synthesis. (TIF)

S5 Table B_i_ values for CN1, CN2, and CN3. (TIF)

S6 Table C_n_ values for CN1, CN2, and CN3. (TIF)

S7 Table β*i* values for CN1, CN2, and CN3. (TIF)

S1 Data Gene expression. The temporal dynamic of gene expression is reported gene by gene and graft by graft. In the main Excel sheet “All Genes_All Cluster” column A and B individuate the IPA symbol for the single gene, column C indicates the cluster the gene belongs to, columns D-G mark if the gene impact a specific cellular events, column H indicates the area of activity of the gene, columns I-L correspond to different grafts tested in absence of flow, columns M-BS correspond to the gene expression level for the single graft, at the specific time point in a specific shear condition among the ones previously described. The sheet “All Genes_All Clusters_SORTED 1” corresponds to the list of genes sorted by clusters and with mean values associated. The sheet “MeanTrend_ALLClusters_SORTED1” corresponds to the mean trend of all clusters. The sheet “Mean Trend ALL cluster sorted2” corresponds to the mean trend of all clusters sorted by cellular events of impact.
(XLSX)

S2 Data Graft morphology. The temporal dynamic of lumen, IEL, and EEL radius and intimal, medial thickness is recorded at the time of implantation (t = 0) and after 2 hours, 1,3,7,14,28 days along with the Flow rate, Shear Stress and Tension values. The sheet “Compiled List of VG Morphology” includes the list of all the grafts used for the analysis, the sheet “Sorted List” includes the list of all the grafts used for the analysis with the mean values of all the significant biological measurements, and the sheet “Final tables” includes the mean values of all the significant biological measures under high, low, and intermediate shear along with their correspondent temporal plots.
(XLS)

S3 Data Cellular events temporal dynamic. The temporal dynamic of the cellular events is reported in the correspondent Excel sheet. Each event is tracked at 0, 0.08 hours and 1,3,7,14,28 days through different biological measures and under low, high, and intermediate shear stress conditions: i) Proliferation Rate and ii) Apoptosis Rate is evaluated by measuring the mitotic/apoptotic density in cells/mm^2^; iii) Matrix Growth Rate is evaluated by measuring the Matrix Area Change Rate in mm^2^/day; iv) EEL Growth Rate (cellular movement) is evaluated by measuring the EEL Area Rate of Change in mm^2^/day.
(XLS)
