# Supplementary figures and images for: Linking gene dynamics to vascular hyperplasia – Toward a predictive model of vein graft adaptation
Source: PLoS One. 2017 Nov 30;12(11):e0187606. doi: 10.1371/journal.pone.0187606 (PMC5708843; doi:10.1371/journal.pone.0187606)

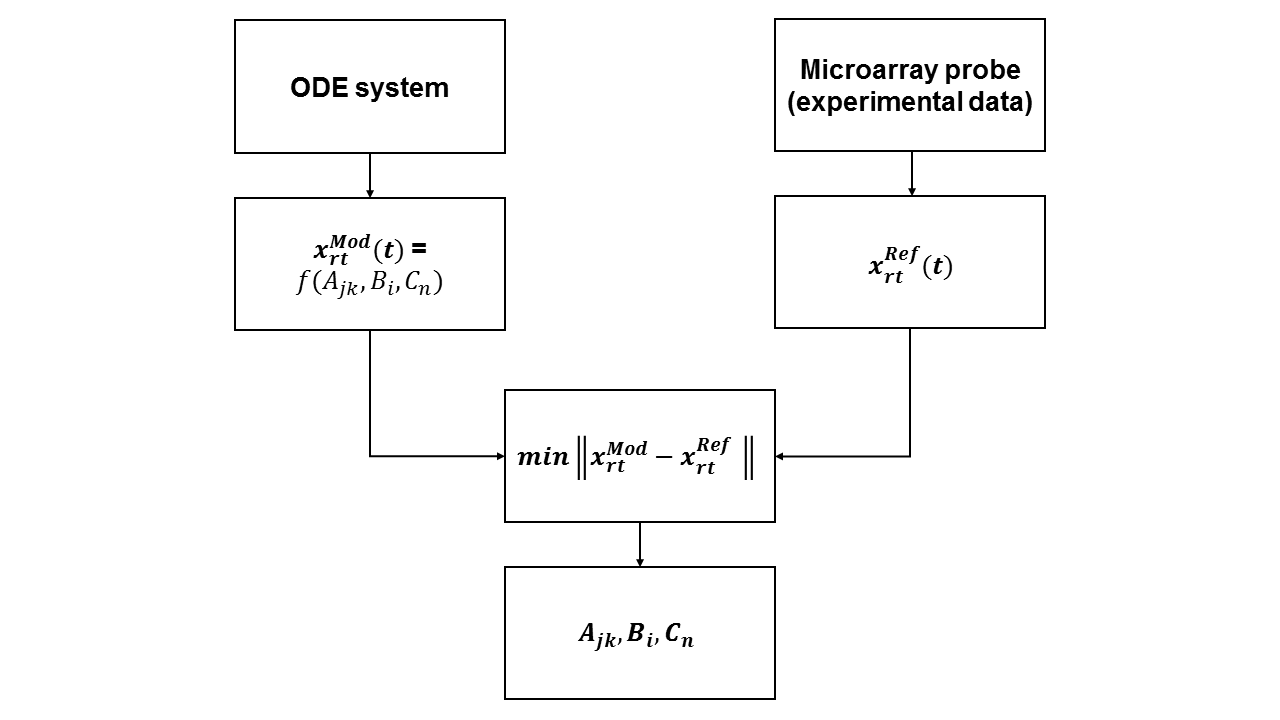

Supplement: S1 Fig — The distance between the mathematical model (replicated with an ODEs system) and the experimental data (from rabbit specific microprobe) is minimized with a GA and the unknowns of the CN are retrieved. (TIF) [file pone.0187606.s001.tif]

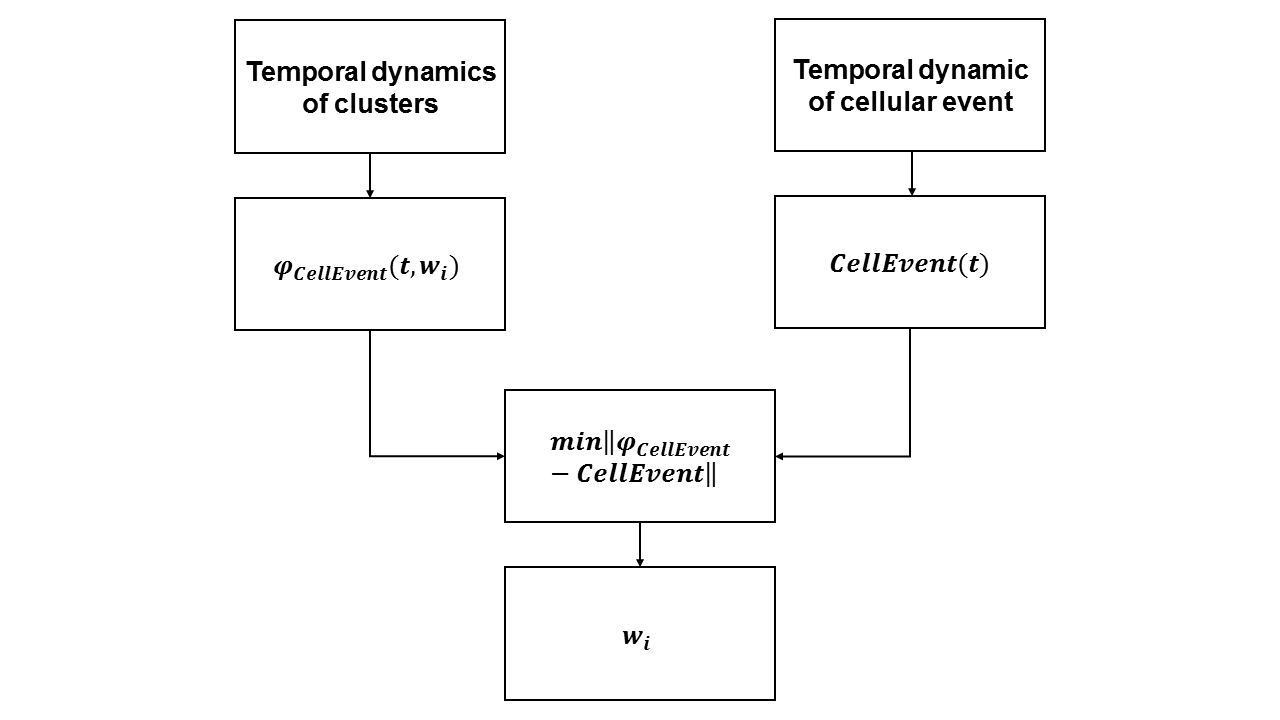

Supplement: S2 Fig — A linear combination of clusters dynamics, mediated with the level of activity that each cluster employs on the cellular event, is fitted on the temporal dynamic of the relative biologic process. (TIF) [file pone.0187606.s002.tif]

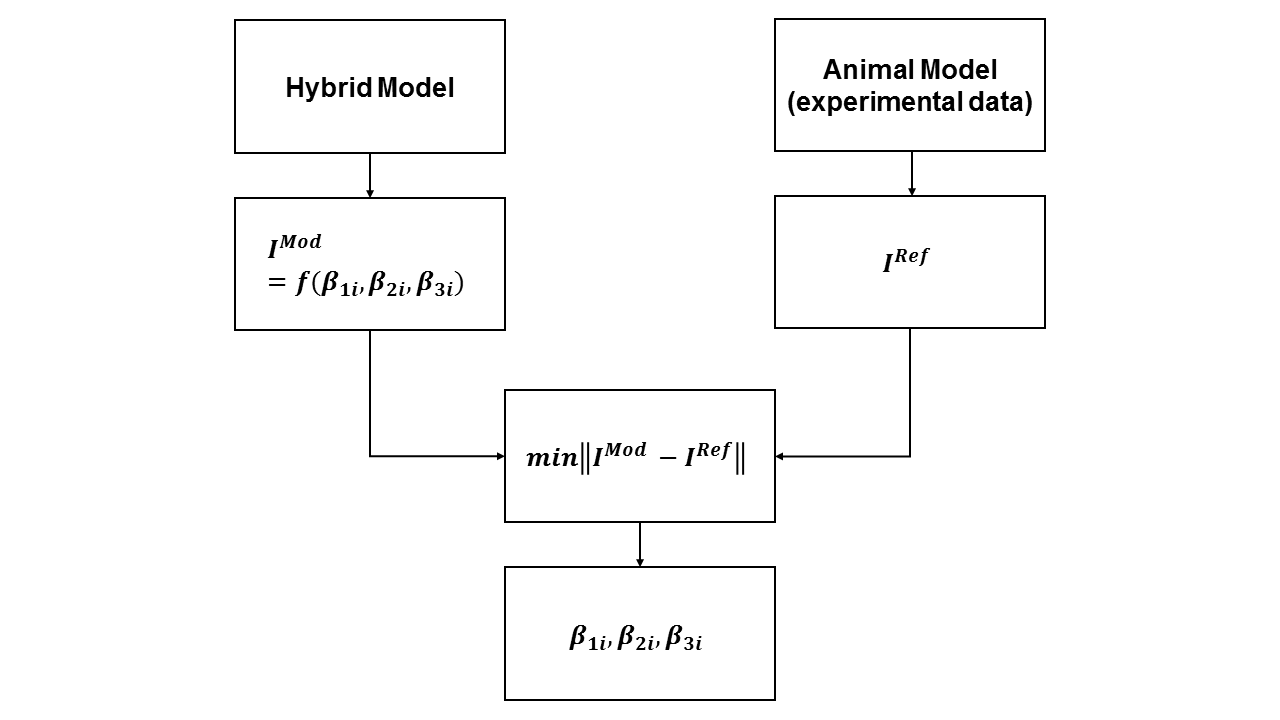

Supplement: S3 Fig — The distance between the intimal thickness dynamic recorded from experimental data and the hybrid model is minimized with a GA and the unknowns of the model are retrieved. (TIF) [file pone.0187606.s003.tif]

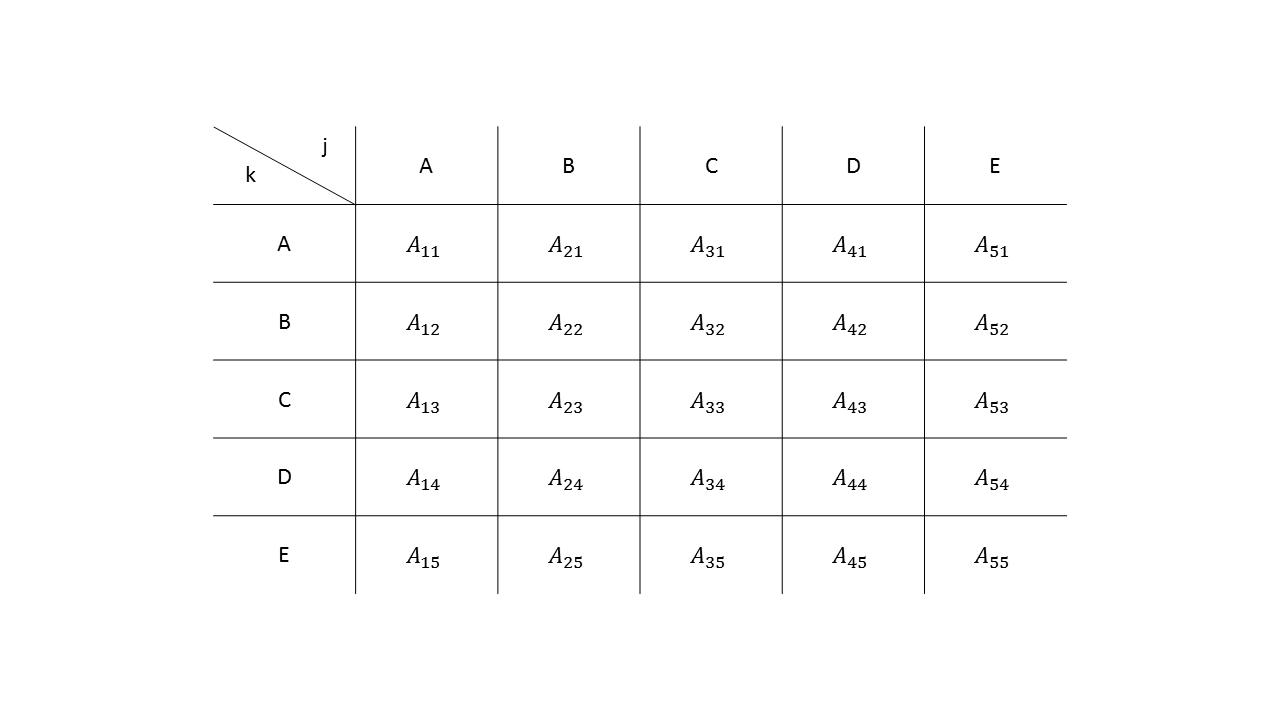

Supplement: S1 Table — Mutual level of interconnectedness among clusters. (TIF) [file pone.0187606.s004.tif]

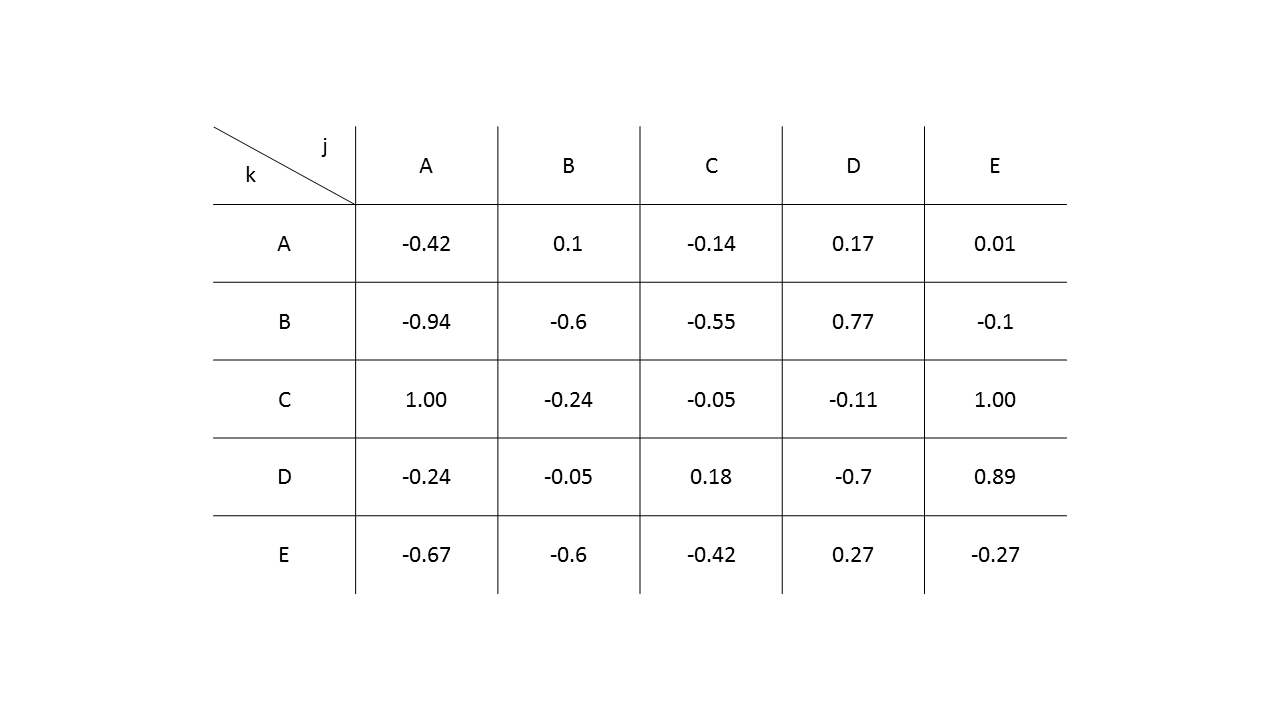

Supplement: S2 Table — (TIF) [file pone.0187606.s005.TIF]

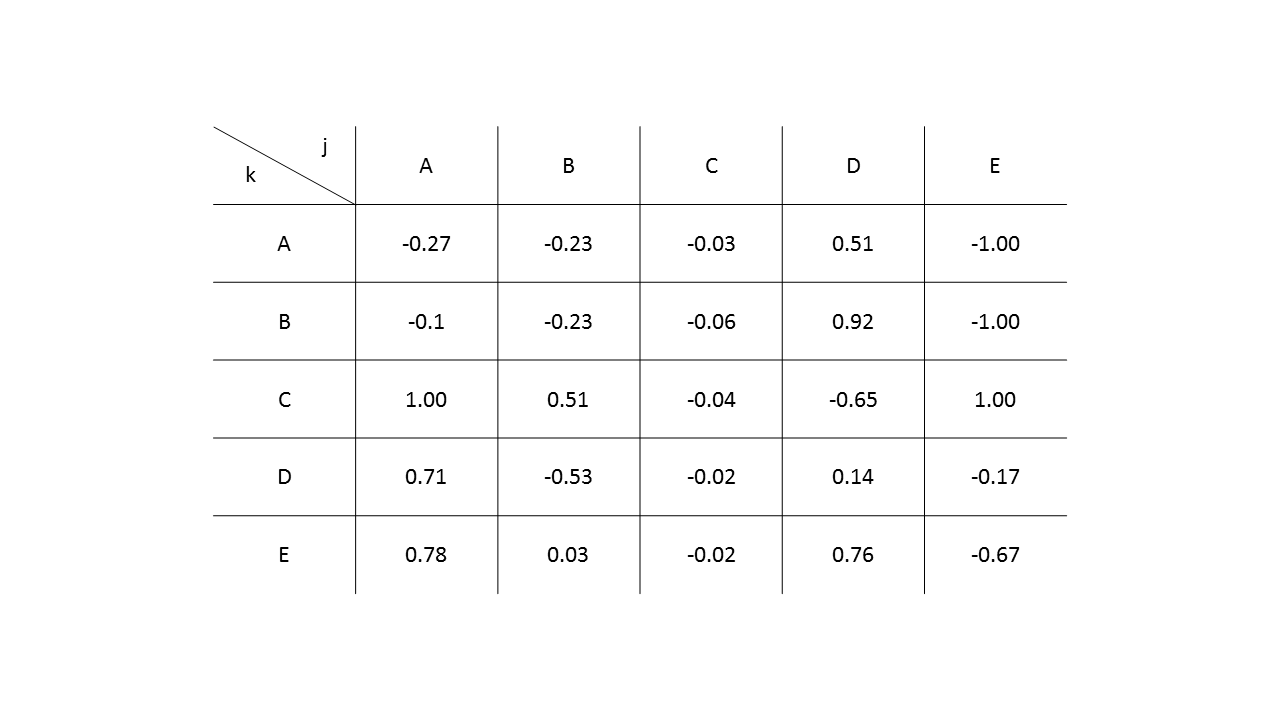

Supplement: S3 Table — (TIF) [file pone.0187606.s006.TIF]

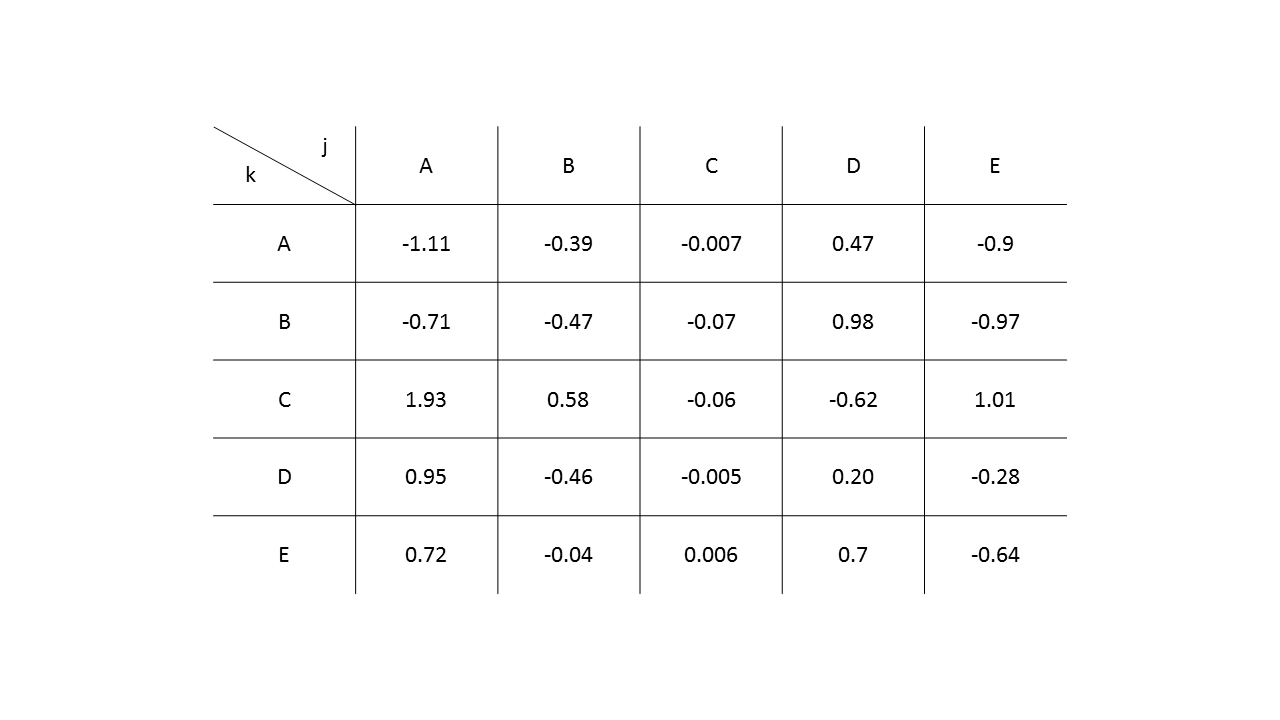

Supplement: S4 Table — (TIF) [file pone.0187606.s007.TIF]

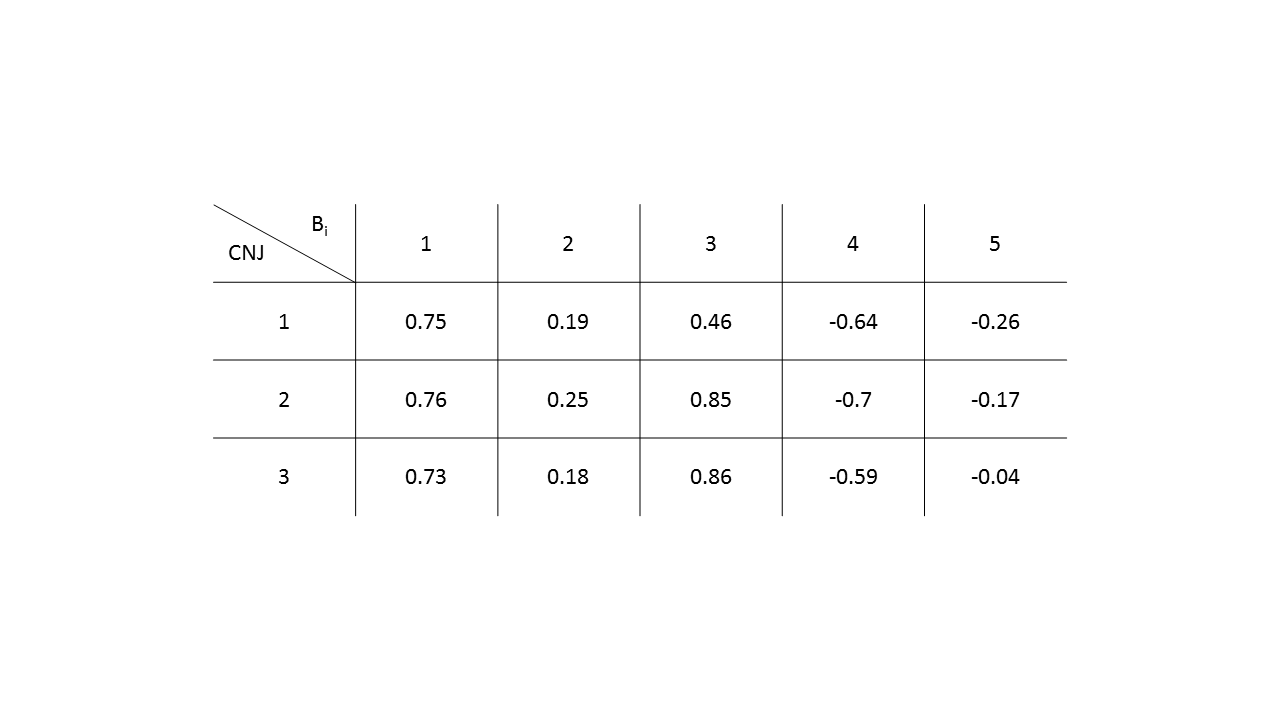

Supplement: S5 Table — (TIF) [file pone.0187606.s008.TIF]

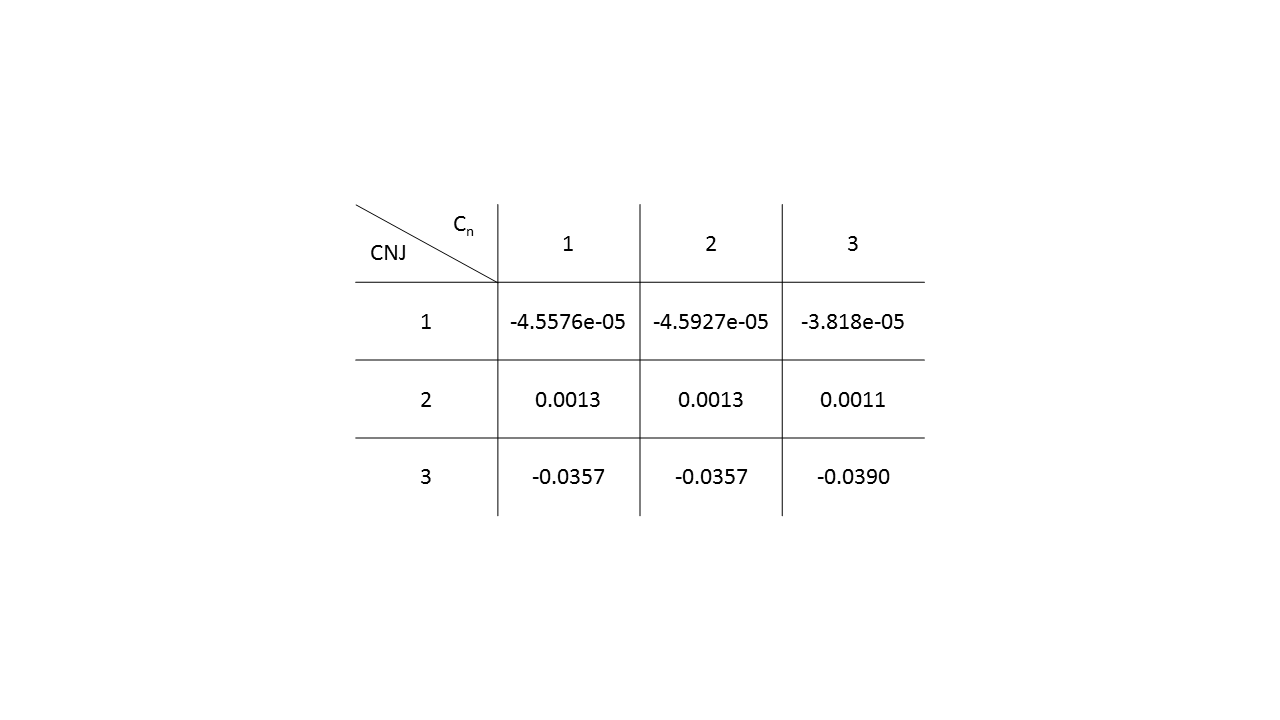

Supplement: S6 Table — (TIF) [file pone.0187606.s009.TIF]

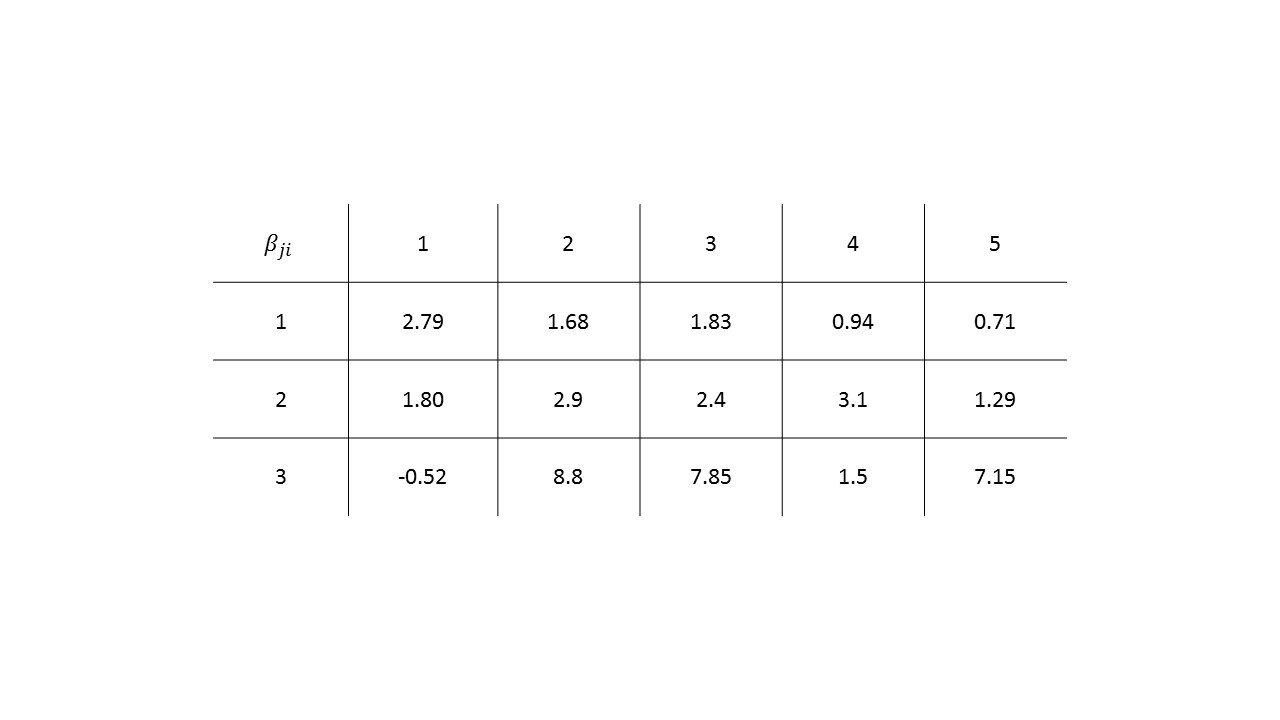

Supplement: S7 Table — (TIF) [file pone.0187606.s010.TIF]
